# Neuroarchitecture of Aminergic Systems in the Larval Ventral Ganglion of *Drosophila melanogaster*

**DOI:** 10.1371/journal.pone.0001848

**Published:** 2008-03-26

**Authors:** Matthias Vömel, Christian Wegener

**Affiliations:** Emmy Noether Neuropeptide Group, Animal Physiology, Department of Biology, Philipps-University Marburg, Marburg, Germany; Emory University, United States of America

## Abstract

Biogenic amines are important signaling molecules in the central nervous system of both vertebrates and invertebrates. In the fruit fly *Drosophila melanogaster*, biogenic amines take part in the regulation of various vital physiological processes such as feeding, learning/memory, locomotion, sexual behavior, and sleep/arousal. Consequently, several morphological studies have analyzed the distribution of aminergic neurons in the CNS. Previous descriptions, however, did not determine the exact spatial location of aminergic neurite arborizations within the neuropil. The release sites and pre-/postsynaptic compartments of aminergic neurons also remained largely unidentified. We here used *gal4*-driven marker gene expression and immunocytochemistry to map presumed serotonergic (5-HT), dopaminergic, and tyraminergic/octopaminergic neurons in the thoracic and abdominal neuromeres of the *Drosophila* larval ventral ganglion relying on Fasciclin2-immunoreactive tracts as three-dimensional landmarks. With tyrosine hydroxylase- (TH) or tyrosine decarboxylase 2 (TDC2)-specific *gal4*-drivers, we also analyzed the distribution of ectopically expressed neuronal compartment markers in presumptive dopaminergic TH and tyraminergic/octopaminergic TDC2 neurons, respectively. Our results suggest that thoracic and abdominal 5-HT and TH neurons are exclusively interneurons whereas most TDC2 neurons are efferent. 5-HT and TH neurons are ideally positioned to integrate sensory information and to modulate neuronal transmission within the ventral ganglion, while most TDC2 neurons appear to act peripherally. In contrast to 5-HT neurons, TH and TDC2 neurons each comprise morphologically different neuron subsets with separated in- and output compartments in specific neuropil regions. The three-dimensional mapping of aminergic neurons now facilitates the identification of neuronal network contacts and co-localized signaling molecules, as exemplified for DOPA decarboxylase-synthesizing neurons that co-express crustacean cardioactive peptide and myoinhibiting peptides.

## Introduction

Biogenic amines (BAs) originate from the amino acid metabolism and act as important intercellular signaling molecules in both vertebrates and invertebrates. In insects, BAs are often synthesized in interneurons and serve a neuromodulator/-transmitter function within the CNS (see [1], [2]). Several efferent neurons also release BAs as neurohormones at peripheral targets like the body wall muscles (see [3]). In *Drosophila melanogaster*, BAs play vital roles in the modulation of many physiological processes such as feeding, learning/memory, locomotor activity, sexual behavior, and sleep/arousal (see e.g. [1], [2], [4]). However, the specific cellular targets of aminergic neurons largely remain unidentified. The small size and chemical structure of BAs complicates antibody production. BA labeling also typically depends on specific fixation procedures that can impair the simultaneous detection of other proteins in co-labeling experiments. To circumvent these technical difficulties, several studies have focused on the detection of enzymes catalyzing BA production or degradation.

Available morphological studies primarily describe the position of aminergic neuron somata within the CNS, but usually do not characterize finer neuronal projections. Data on the accurate spatial distribution of most BA receptors within the CNS are missing as well. Out of 21 BA receptors identified *in silico* (see [5], [6]), only three dopamine ([7]–[10]; see [11]) and three serotonin receptors [12]–[14], and one octopamine receptor ([15]; see [16]) have been localized on a cellular level. Besides this lack of information about the cellular targets of specific aminergic neurons, nothing is known about neurons providing synaptic input to aminergic neurons in *Drosophila*.

We therefore set out to provide a three-dimensional morphological characterization of aminergic neurons in the thoracic and abdominal neuromeres of the *Drosophila* larval ventral ganglion (VG). The VG is particularly suited for the analysis of aminergic networks since it offers several advantages: 1) The VG contains both aminergic interneurons and efferent neurons, but has a simpler architecture and fewer neurons than the brain (see [2]). Nevertheless, the VG receives various sensory inputs and takes part in the regulation of essential physiological processes such as locomotion (see [17]) and ecdysis (see [18]). 2) Several light-optical microscopy studies already described the distribution of aminergic neurons and enzymes catalyzing BA production/degradation. Thus, the total number and soma position of aminergic neurons in the VG is known (see [1]). 3) A set of evenly distributed landmarks permits the accurate three-dimensional charting of aminergic projections within the VG neuropil [19]. These landmarks, which are labeled by antibodies against the cell adhesion molecule Fasciclin2 (Fas2), remain constant between larvae and throughout the larval stages, and recently served as anatomical coordinates to map motoneurons [19], peptidergic [20] and sensory neurons [21], [22].

Our three-dimensional maps of presumed serotonergic, dopaminergic and tyraminergic/octopaminergic neurons allow the tracing of single aminergic neurites to defined neuropil areas within the VG. Thus, the maps facilitate the comparison of aminergic neuron morphology with e.g. a specific BA receptor expression pattern. We also analyzed the distribution of ectopically expressed neuronal compartment markers in presumed dopaminergic and tyraminergic/octopaminergic neurons. The respective markers indicated distinct in- and output sites, which can now be compared with high spatial resolution to other charted neuronal projections. Finally, our mapping helps to identify co-localized signaling molecules and receptors in aminergic neurons, and improves the suitability of aminergic neurons as anatomical landmarks within the VG.

## Methods

### Fly stocks

Wild-type Oregon R (OrR) flies and progeny from crosses of appropriate *gal4* driver lines with UAS-marker gene lines [23] were used for immunocytochemistry (for detailed information on the employed fly lines see references in Table 1). All flies were reared under a 12:12 h light:dark regime at 18 or 25°C on standard cornmeal agar medium and yeast.

**Table 1:** Employed fly lines.

Fly Line	Donor [Reference]
*Ccap-gal4*	John Ewer [111]
*Ddc-gal4*	Bloomington Stock Center; Jay Hirsh [56]
*Tdc2-gal4*	Bloomington Stock Center; Jay Hirsh [60]
*Th-gal4*	Bloomington Stock Center; Serge Birman [49]
*UAS-DscamGFP*	Tzumin Lee [53]
*UAS-mCD8GFP*	Bloomington Stock Center; Tzumin Lee and Liqun Luo [112]
*UAS-SybGFP*	Bloomington Stock Center; Kendal Broadie [50], [51]
*UAS-SytGFP*	Bloomington Stock Center; Kendal Broadie [52]

### Immunocytochemistry

For whole-mount immunostainings, CNS of third instar (L3) larvae were dissected in ice-cold 0.1 M sodium phosphate buffered saline (PBS; pH 7.2) and fixed with 4 % paraformaldehyde in PBS for 2 h at 4°C. After several washes with 0.1 M PBS containing 1 % TritonX (PBT), tissue was incubated with primary antibody (for detailed information on the employed antisera see references in Table 2) and 5 % normal goat serum (NGS) in PBT for 1–3 days at 4°C. The tissue was then washed several times with PBT, followed by overnight incubation with the appropriate secondary antibodies and 1 % NGS in PBT at 4°C. As secondary antibodies, Cy3-conjugated AffiniPure goat-anti-rabbit and goat-anti-rat IgG, and Cy5-conjugated AffiniPure goat-anti-rabbit and goat-anti-mouse IgG (Jackson ImmunoResearch, West Grove, PA) were used at a dilution of 1∶300. The following day, tissue was washed several times with PBT, twice with 0.01 M PBS, and then mounted in glycerol:0.01 M PBS (80∶20). To visualize fine neurites and to enhance figure contrast, *gal4*-driven expression of mCD8GFP or GFP-tagged fusion proteins like SybGFP was generally revealed with a commercially available polyclonal rabbit-anti-GFP serum (MBL International, Woburn, MA). This GFP antiserum specifically labeled GFP expressing neurons (Fig. S1).

**Table 2:** Employed antisera.

Antibody	Dilution	Source	Donor [Reference]
anti-5-HT polyclonal	1∶5000	rabbit	Immunostar Inc., Hudson, WI
anti-CCAP polyclonal	1∶1000	rabbit	Heinrich Dircksen [111], [113], [114]
anti-DDC polyclonal	1∶5000	rabbit	Ross Hodgetts [55]
anti-Fas2 1D4 monoclonal	1∶75	mouse	Developmental Studies Hybridoma Bank, University of Iowa, IA
anti-GFP polyclonal	1∶500	rabbit	MBL International, Woburn, MA
anti-TβH polyclonal	1∶100	rat	Maria Monastirioti [61]
anti-TH monoclonal	1∶1000	mouse	Immunostar Inc., Hudson, WI
anti-MIP	1∶3000	rabbit	Manfred Eckert [115]

### Confocal microscopy and data processing

Confocal stacks were acquired on a confocal laser scanning microscope (Leica TCS SP2, Leica Microsystems, Wetzlar, Germany) with a 40x objective (HCX PL APO 40x, N.A. 1.25) at 512×512 pixel resolution in 0.5–1 µm steps along the z-axis. For 3D volume-rendering, image stacks were imported into AMIRA 3.1 software (Indeed-Visual Concepts, Berlin, Germany) and processed using the “Voltex” and “ObliqueSlice” module. A false color map was applied to the volume-rendered neurons, and brightness and contrast were adjusted. Snapshots were taken in AMIRA and processed with Adobe Photoshop 7.0 (Adobe Systems Inc., San Jose, CA). A schematic representation of the BA labeling in the Fas2 landmark system was generated using Adobe Illustrator CS 11.0 (Adobe Systems Inc., San Jose, CA). To analyze co-localization between *gal4*-driven mCD8GFP expression and immunolabeling, volume-rendered 3D image stacks were down-sampled to maximum pixel intensity projections and further processed with Adobe Photoshop 7.0 and ImageJ 1.34s (NIH, public domain: http://rsb.info.nih.gov/ij/).

## Results

**Figure 1:**
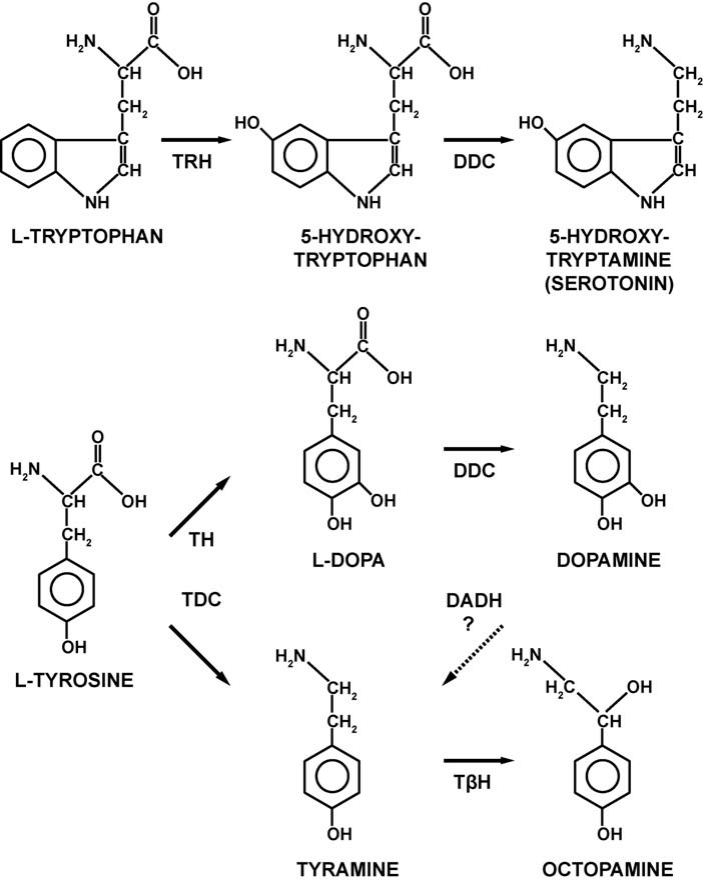
Synthetic pathways for serotonin, dopamine, tyramine, and octopamine. The amino acid tryptophan is the starting point for the synthesis of 5-hydroxytryptamine (5-HT, serotonin), whereas tyrosine is the source for dopamine as well as tyramine and octopamine. An alternative pathway (dotted arrow) via DADH may exist that permits the synthesis of tyramine from dopamine. DADH: dopamine dehydroxylase; DDC: DOPA decarboxylase; TβH: tyramine β-hydroxylase; TDC: tyrosine decarboxylase; TH: tyrosine hydroxylase; TRH: tryptophan hydroxylase.

**Figure 2:**
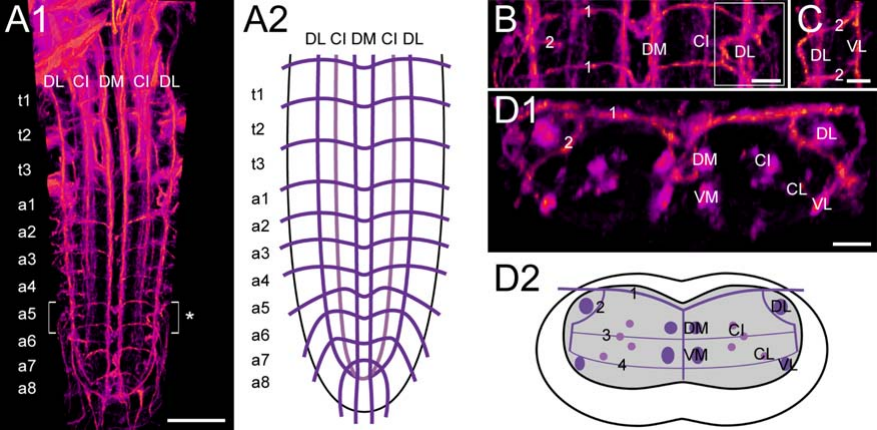
The Fasciclin2 landmark system in the *Drosophila* larval VG. A1) Dorsal view of a 3D image stack showing volume-rendered Fas2-immunoreactive tracts in the thoracic (t1-3) and abdominal neuromeres (a1-8) of the L3 larval VG, and A2) the deduced idealized dorsal scheme. B) Detailed dorsal and C) lateral view of a5 and adjacent neuromeres (* in A1). D1) Transversal view of a5, and D2) the deduced idealized transversal scheme. Longitudinal projections are named according to their relative dorso-ventral (D: dorsal, C: central, V: ventral) and medio-lateral (M: medial, I: intermedial, L: lateral) position. For example, the two dorso-medial Fas2-immunoreactive tracts are referred to as DM tracts. Transversal projections (TP) are numbered according to their relative dorso-ventral position, i.e. “1” represents the topmost TP [19]. Scale bars: 50 µm in A), 10 µm in B), C), and D).

We used targeted fluorescent marker protein expression [23] and immunocytochemistry to map aminergic neurons in the thoracic and abdominal neuromeres of the *Drosophila* ventral ganglion (referred to as VG; the subesophageal neuromeres are ignored here since they show a less strict Fas2 labeling pattern than the thoracic and abdominal neuromeres) at the L3 larval stage. Since the immunocytochemical detection of most BAs is complicated (see [24]) we mainly focused on the enzymes involved in BA biosynthesis (for an overview about BA biosynthesis see Fig. 1). Consequently, our data do not chemically proof the presence of BAs in the characterized neurons, but indicate that the respective neurons are able to synthesize BAs. Thus, we mapped presumed serotonergic, dopaminergic, and tyraminergic/octopaminergic neurons into a set of constant and evenly distributed landmarks of the VG that show Fas2 immunoreactivity (for a description of the Fas2 landmark system see Figure 2). Since we could not simultaneously label histamine and Fas2, histaminergic neurons are omitted in our mapping (see [1]). We adopted the neutral nomenclature of Fas2-positive tracts proposed by Landgraf et al. ([19]; see Figure 2) and present our results on standardized plates. Each plate comprises both dorsal/ventral and transverse views of a typical whole-mount preparation and schematic drawings showing the characteristic distribution of the respective aminergic neurons within the Fas2 landmark system. The corresponding text section contains a detailed morphological description as well as a short overview of the synthesis pathway of the respective BA, its physiological functions, and references to previous morphological studies. In case appropriate *gal4* drivers were available, we also analyzed the distribution of ectopically expressed pre- and postsynaptic markers to reveal putative synaptic in-/output zones of aminergic neurons.

### 5-HT neurons

**General characteristics.** In *Drosophila*, the tryptophan hydroxylases DTPH (encoded by CG7399) and DTRHn (encoded by CG9122) catalyze the conversion of tryptophan to 5-hydroxytryptophan [25]–[27]. DOPA decarboxylase may then metabolize 5-hydroxytryptophan to 5-hydroxytryptamine (5-HT, serotonin). In the larva, 5-HT containing neurons influence endocrine activity, feeding (see [1]), and heart rate [28].

Previous morphological descriptions of 5-HT producing neurons in the larval VG relied on immunostainings [29]–[31]. We used a commercially available rabbit-anti-5-HT polyclonal antibody (Immunostar Inc.) and observed a staining pattern identical to those previously described.

**Figure 3:**
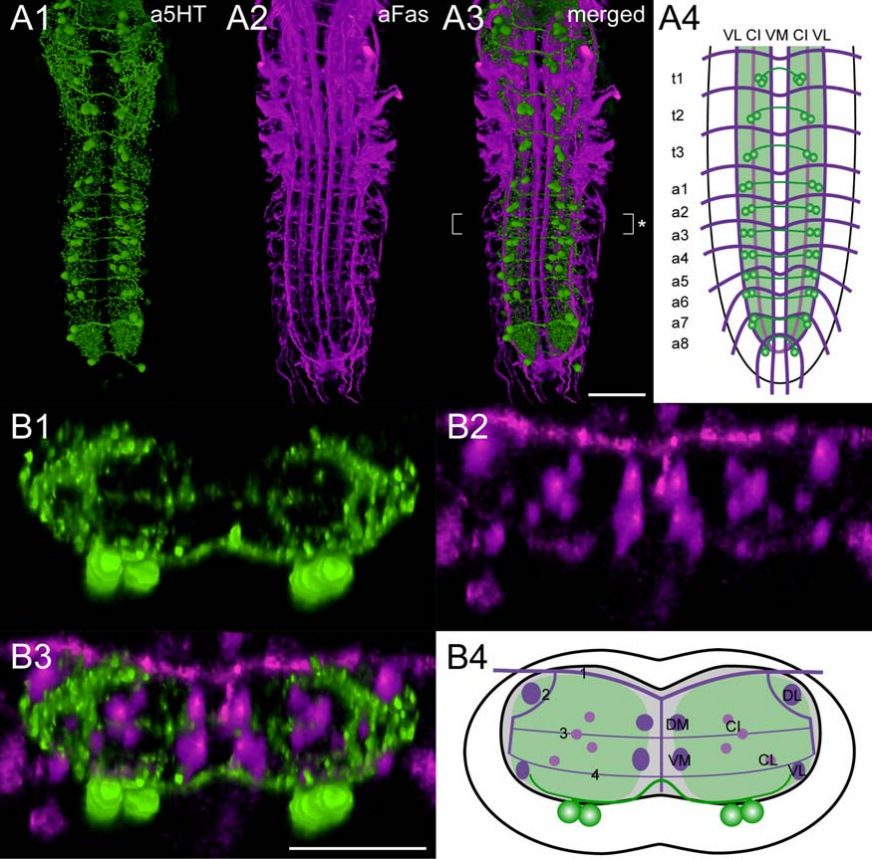
Mapping of 5-HT-immunoreactive neurons. Ventral view of 3D image stacks showing volume-rendered A1) 5-HT-immunoreactive neurons (green) and A2) Fas2-positive tracts (magenta) in a larval VG. The merged image (A3) served for an idealized schematic representation of presumed 5-HT neurons in the Fas2 landmark system (A4). B1-3) Corresponding transversal views of the neuromere a2 (* in A3), and B4) the deduced idealized transversal scheme. Green-shaded areas in the schemes contain a high concentration of 5-HT-immunoreactive arborizations. Scale bars: 50 µm in A), 25 µm in B).

**Morphology** (Fig. 3). The VG contains segmentally reiterated bilateral pairs of 5-HT-immunoreactive neurons (5-HT neurons). Typically, two 5-HT neuron pairs reside in each of the thoracic and abdominal neuromeres, with exception of t1 (three pairs) and a8 (one pair). While the somata of thoracic 5-HT neurons reside ventro-medially in the cortex beneath the CI tracts, the somata of abdominal 5-HT neurons lay rather ventro-laterally beneath the VL tracts. In each neuromere, 5-HT neurons project below the transversal projection (TP) 4 ventro-medially until their neurites pass the midline beneath the VM tracts. On the contralateral side, 5-HT neurites bifurcate strongly and innervate the whole neuropil. Thus, we could not trace single 5-HT neurites to separate target sites. Other 5-HT neurites run at the height of TP 3 between the DM and VM tracts and appear to connect the bilateral neuropil parts. Compared to other neuromeres, a7 showed a particularly high concentration of 5-HT neurites. In contrast, a8 and the adjacent “terminal plexus” [19] lacked 5-HT neurite arborizations. The 5-HT neurons of a8 send their primary neurites to the contralateral side, and then appear to supply the neuropil of a7.

### Tyrosine hydroxylase neurons

**General characteristics.** Dopamine (DA) synthesis depends on the concerted action of the enzymes tyrosine hydroxylase (TH) and DOPA decarboxylase. TH catalyzes the first and rate-limiting step in catecholamine biosynthesis and mediates the oxidation of tyrosine to 3,4-dihydroxy-L-phenylalanine (L-DOPA). DOPA decarboxylase may then metabolize L-DOPA to DA. In *Drosophila*, DA plays a role in various complex neuronal processes such as sleep and arousal [32]–[34], visual attention [35], stress response [36], learning [37], and sexual behavior [38]. In the larval and adult CNS, DA and TH immunoreactivity appear to localize to the same neurons [29], [39], [40]. Thus, TH-immunoreactive neurons are commonly referred to as dopaminergic neurons (see [1]). Central TH neurons specifically synthesize only one out of two possible TH splice variants [41], [42] from a primary transcript encoded by the *pale* locus (CG10118; [43], [44]). The second TH splice variant locates to epidermal cells and serves a vital role in cuticle biosynthesis [45]. Genetic as well as pharmacological inhibition of TH activity suggests that catecholamine loss decreases locomotor activity [46], [47].

Previous studies described the morphology of TH-producing neurons (TH neurons) in the VG with immunocytochemistry [29], [48] and *Th-gal4*-driven GFP expression [49]. We here used the same *Th-gal4* driver line as well as a commercially available monoclonal mouse-anti-TH antibody. In general, both approaches revealed identical neurons. Ventral midline neurons in a1-7, however, showed very weak or even lacked *Th-gal4*-driven mCD8-GFP expression (Fig. S1), but always immunostained against TH (Fig. S2).

**Figure 4:**
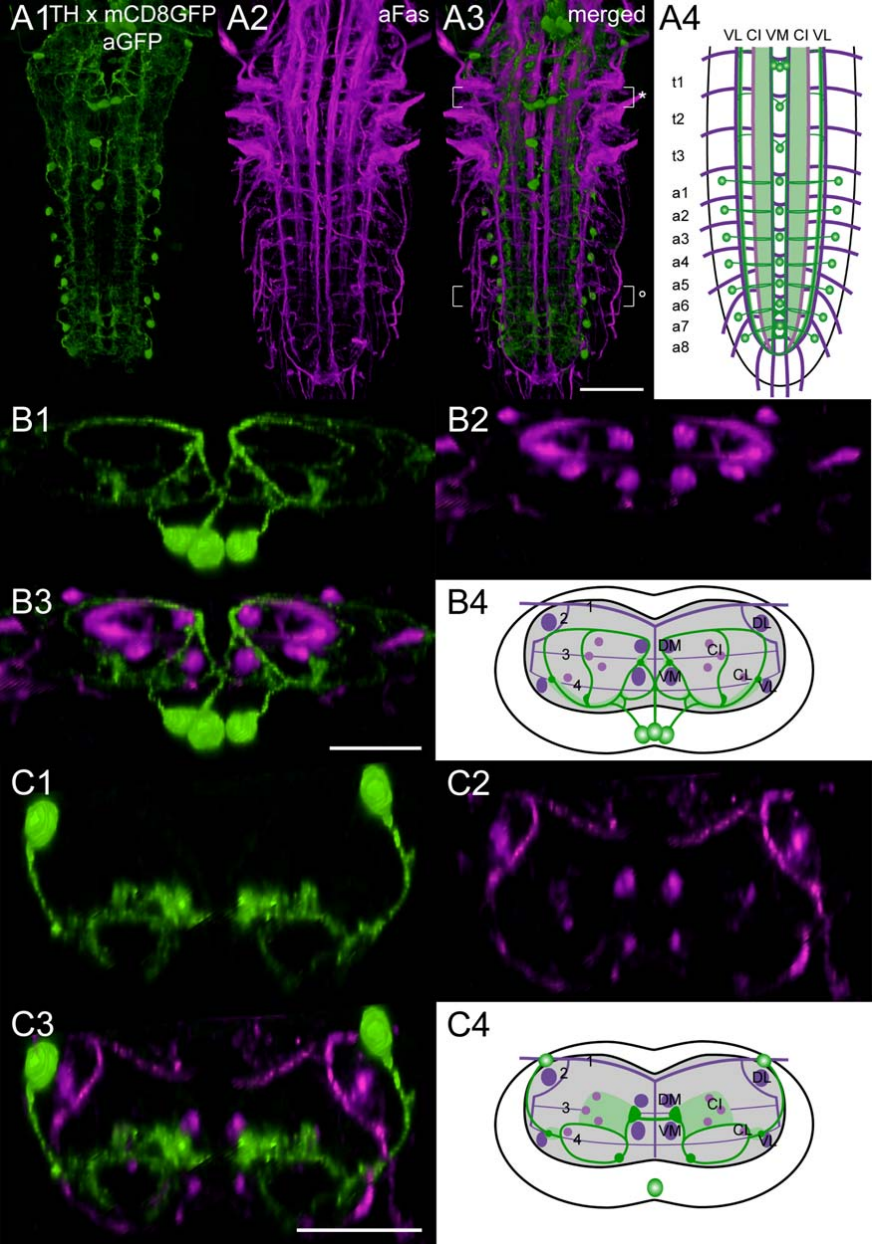
Mapping of *Th-gal4* x mCD8GFP expressing neurons. A1) Ventral view of 3D image stacks showing volume-rendered GFP-immunoreactive neurons (green) and A2) Fas2-positive tracts (magenta) in a larva expressing *Th-gal4*-driven mCD8GFP. The merged image (A3) served for an idealized schematic representation of *Th-gal4* x mCD8GFP expressing neurons in the Fas2 landmark system (A4). B1-3) Corresponding transversal views of the neuromere t1 (* in A3), and B4) the deduced idealized scheme. C1-3) Transversal views, and C4) idealized scheme of a5 (° in A3). Note that the idealized schemes A4) and C4) include the vmTH neurons of a1-7, which often lacked *Th-gal4*-driven mCD8GFP expression. These neurons, however, always showed strong TH immunoreactivity (see Fig. S2). Green-shaded areas in the schemes contain a high concentration of GFP-immunoreactive arborizations. Scale bars: 50 µm in A), 25 µm in B) and C).

**Morphology** (Fig. 4). Inferred from *Th-gal4*-driven marker gene expression as well as TH immunostainings, the VG contains two morphologically different TH neuron groups: The first group comprises three ventral median TH neurons (vmTH neurons) in t1, and a single vmTH neuron in each neuromere from t2 to a7. Their cell bodies locate to the midline beneath the VM tracts. The second TH neuron group consists of a bilateral pair of dorso-lateral TH neurons (dlTH neurons) with somata residing at the height of the DL tracts in each neuromere from a1-7. Longitudinal TH projections are adjacent to the VL, beneath the CI, and close to the VM/DM tracts. Neurites of the vmTH neurons project dorsally and then appear to join longitudinal TH projections between the DM and VM tracts. The vmTH neurons of t1 also initially project dorsally until their neurites reach the height of the VM tracts. The neurites then diverge and build up a loop enclosing the DM/VM and CI tracts on each side of the neuromere. These neurite loops seem to establish a transversal connection between all longitudinal TH projections within the VG. As opposed to the vmTH neurites, the neurites of the dlTH neurons run ventrally and form fine longitudinal projections along the VL tracts. There, TH neurites divide and proceed in a loop to the median neuropil. Between the CI and the VM tracts, the TH neurites running beneath TP 4 join bilateral fine longitudinal projections somewhat ventro-laterally to the VM tracts. The TH neurites then proceed dorsally until they converge with the upper branch of the TH neurite loop in a prominent longitudinal projection between the DM and VM tracts. TH neurites ramify heavily in the neuropil between the DM/VM and the CI tracts.

**Figure 5:**
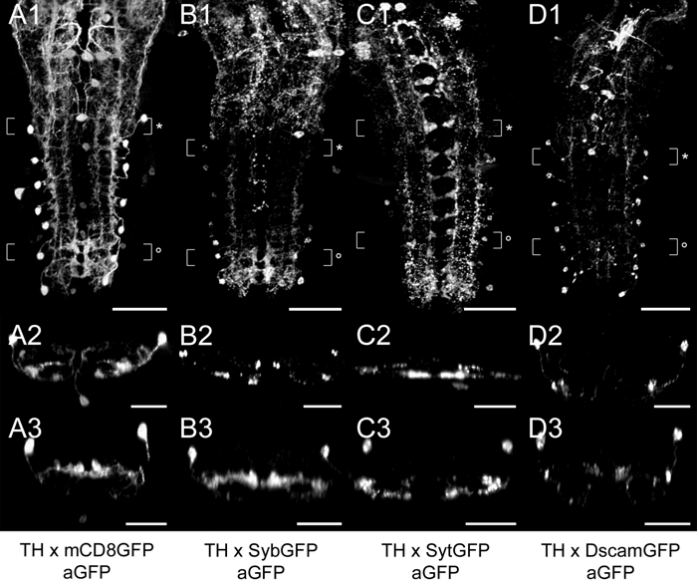
Distribution of ectopically expressed neuronal compartment markers in TH neurons. A1-D1) Dorsal view of GFP immunoreactivity in the VG of larvae expressing *Th-gal4*-driven mCD8GFP (A1-3), SybGFP (B1-3), SytGFP (C1-3), or DscamGFP (D1-3). A2-D2) Corresponding transversal views of the neuromeres t3 or a1 (* in A1-D1). A3-D3) Additional transversal views showing the compartment marker distribution in a5/6 (° in A1-D1). All images are auto-contrasted maximum pixel intensity projections of volume-rendered 3D image stacks. Scale bars: 50 µm in A1-D1), 25 µm in A2-D2) and A3-D3).

**Distribution of ectopically expressed markers** (Fig. 5). To identify the in- and output compartments of TH neurons, we ectopically expressed the neuronal compartment markers neuronal synaptobrevin-GFP (SybGFP; [50], [51]), synaptotagmin 1-GFP (SytGFP; [52]), and *Drosophila* Down syndrome adhesion molecule [17.1]-GFP (DscamGFP; [53]). *Th-gal4*-driven SybGFP showed a dotted distribution within the VG and largely mimicked the mCD8GFP expression pattern. In t1-3, SybGFP uniformly labeled all TH neurites. The neuromeres a1-5 typically contained less SybGFP than t1-3 and a6-7, since labeling was restricted to TH neuron somata and longitudinal TH projections adjacent to the DM/VM and the VL tracts. Transversal TH neurites appeared to lack SybGFP in a1-5. In a6-7, high amounts of SybGFP accumulated around the DM/VM tracts and also located to transversal TH projections. Particularly, the dorsal branches of the bilateral transversal neurite loops showed intense SybGFP labeling. In contrast to SybGFP, *Th-gal4*-driven SytGFP strongly labeled segmentally reiterated neurite arborizations next to the VM tracts. These median arborizations appeared to belong to the transversal TH neurites connecting both neuropil hemispheres. SytGFP further located to longitudinal projections running along the VL tracts and to the neuropil between the CI and VM tracts. Compared to other neuromeres, a6-7 seemed to contain the highest concentration of SytGFP. There, SytGFP particularly accumulated around the longitudinal TH projections adjacent to the VL tracts and in the ventral branches of the transversal TH neurite loops. *Th-gal4*-driven DscamGFP mainly labeled the somata and primary neurites of the dlTH neurons and the longitudinal TH projections adjacent to the VL tracts. Furthermore, in a6-7, DscamGFP located to longitudinal TH projections next to the DM tracts and to the ventral parts of the transversal neurite loops. The neuropil between the CI and VM tracts, however, showed only faint DscamGFP labeling.

### DOPA decarboxylase neurons

**General characteristics.** DOPA decarboxylase (DDC) catalyzes the production of DA from L-DOPA, and 5-HT from 5-hydroxytryptophan. The *Drosophila Ddc* gene (CG10697) encodes a primary transcript that is alternatively spliced into two distinct DDC isoforms in the CNS and epidermis. DDC mRNA levels and DDC protein activity peak during development at the end of embryogenesis and at the larval, pupal, and adult molts. DDC-producing neurons (DDC neurons) play a vital role in cuticle biosynthesis and other DA- or 5-HT-mediated processes (see [1], [54]).

The distribution of DDC neurons in the VG of *Drosophila* was described with immunocytochemistry [29], [43], [48]. In addition, Landgraf et al. [19] mapped neurons expressing *Ddc-gal4*-driven GFP in the Fas2 landmark system. We revealed DDC neurons with a different polyclonal rabbit-anti-DDC serum [55] as well as *Ddc-gal4*-driven mCD8-GFP [56]. Although the DDC immunolabeling pattern largely matched the *Ddc-gal4*-driven mCD8GFP expression, we observed some substantial differences in the type and number of labeled neurons (Fig. S3).

**Figure 6:**
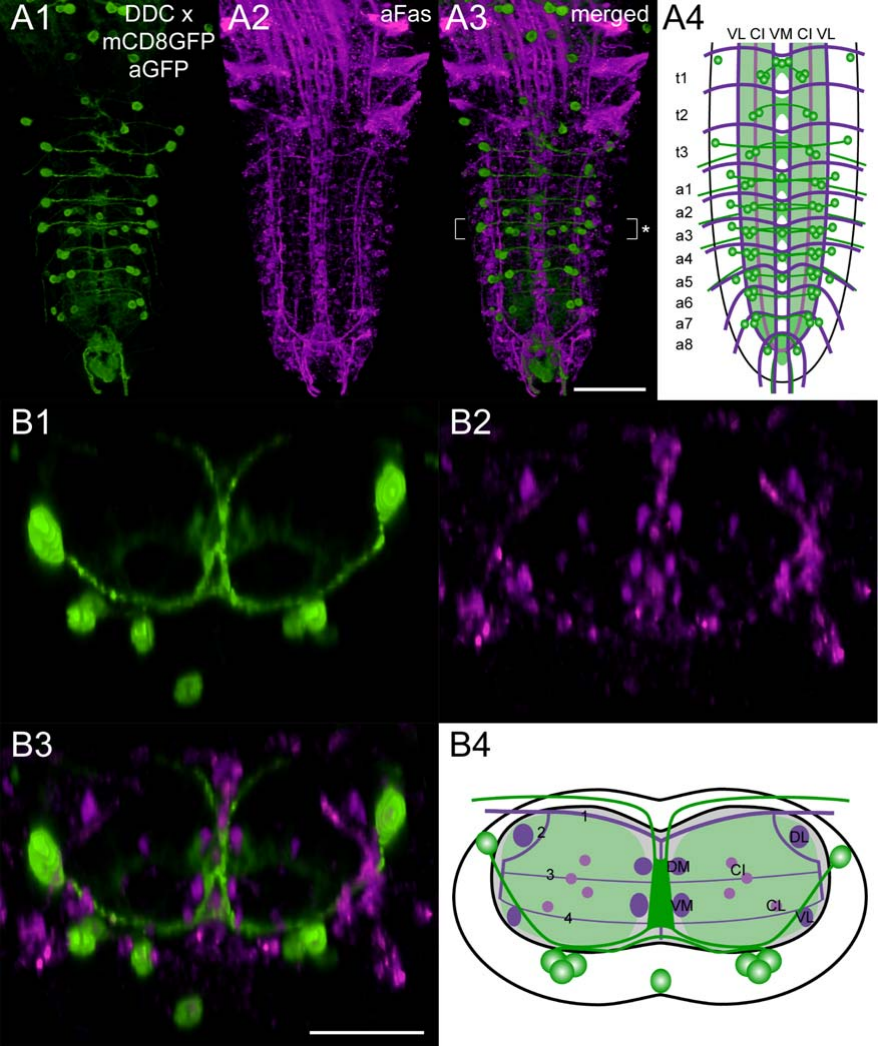
Mapping of *Ddc-gal4* x mCD8GFP expressing neurons. A1) Ventral view of 3D image stacks showing volume-rendered GFP-immunoreactive neurons (green) and A2) Fas2-positive tracts (magenta) in a larva expressing *Ddc-gal4*-driven mCD8GFP. The merged image (A3) served for an idealized schematic representation of *Ddc-gal4* x mCD8GFP expressing neurons in the Fas2 landmark system (A4). B1-3) Corresponding transversal views of the neuromere a4 (* in A3), and B4) the deduced idealized scheme. Note that the *Ddc-gal4* driver typically drives mCD8GFP expression in most, but not all presumed DDC neurons of the VG (see Fig. S3 and S4). Thus, some presumed DDC neurons are not included in the idealized schemes shown here. Green-shaded areas in the schemes contain a high concentration of GFP-immunoreactive arborizations. Scale bars: 50 µm in A), 25 µm in B) and C).

**Figure 7:**
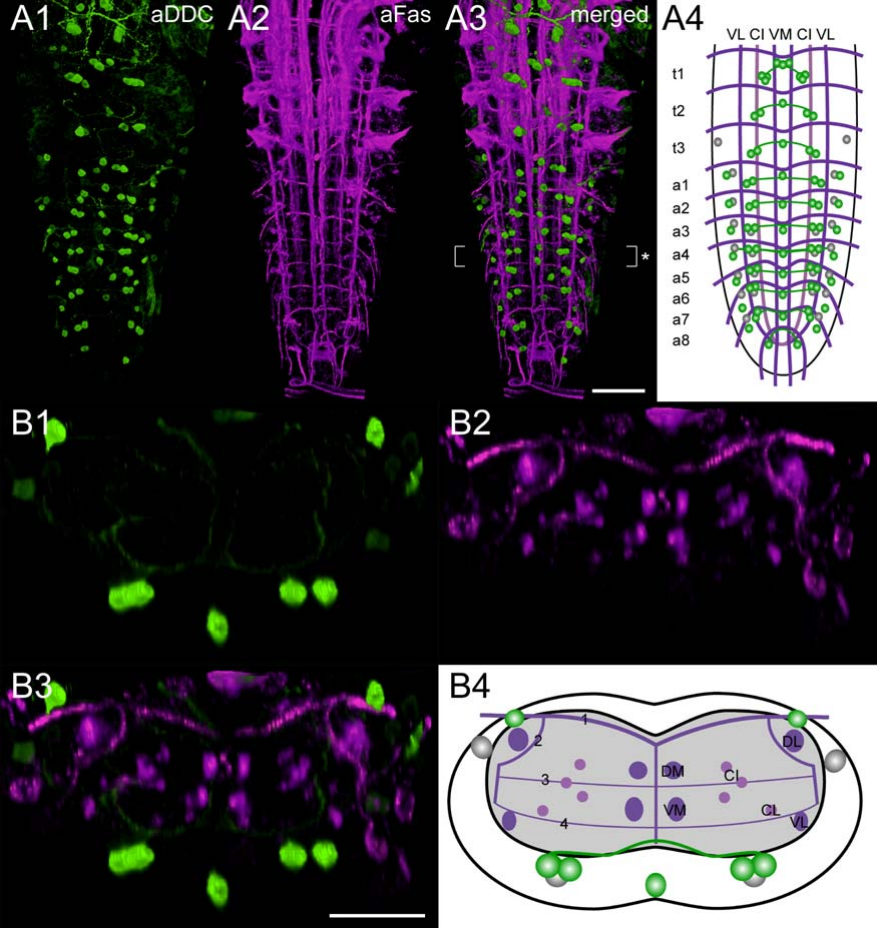
Mapping of DDC-immunoreactive neurons. A1) Ventral view of 3D image stacks showing volume-rendered DDC-immunoreactive neurons (green) and A2) Fas2-positive tracts (magenta) in a larval VG. The merged image (A3) served for an idealized schematic representation of DDC-immunoreactive neurons in the Fas2 landmark system (A4). B1-3) Corresponding transversal views of the neuromere a4 (* in A3), and B4) the deduced idealized scheme. Besides the aminergic neurons, the DDC antiserum also faintly labeled presumed peptidergic neurons, which are shown in gray in the schemes. Noteworthy, these peptidergic neurons are also present in the *Ddc-gal4* expression pattern (see Fig. 6). Scale bars: 50 µm in A), 25 µm in B).

**Morphology** (Fig. 6, 7). Inferred from *Ddc-gal4*-driven marker gene expression (Fig. 6), DDC immunostainings (Fig. 7; Fig. S3), and double labeling experiments with antibodies against 5-HT and TH (Fig. S4), DDC neurons can be categorized into five distinct neuron groups: The first group consists of segmentally reiterated DDC neuron pairs with somata in the ventral cortex below the CI fascicles. These DDC neurons likely represent serotonergic neurons since they showed 5-HT immunoreactivity (Fig. S4; see above for a detailed morphological description). The *Ddc-gal4* driver induced mCD8GFP expression in additional neuron pairs of a3-6 which reside adjacent to the 5-HT neurons in the cortex beneath the CI tracts. These neurons constitute a second distinct DDC neuron group since they obviously synthesize the peptide corazonin [19]. The corazonin-immunoreactive DDC neurons showed faint DDC immunoreactivity in a few preparations, but lacked 5-HT immunoreactivity (Fig. S3, and S4; for a detailed morphological description of corazonin-immunoreactive neurons within the Fas2 landmark system see [20]). The third group of DDC neurons consists of unpaired neurons in the ventral midline of t3 and a1-5, whose somata lay right beneath the DM/VM tracts. Although these neurons typically showed poor *Ddc-gal4*-driven mCD8-GFP expression, they always strongly stained with both DDC and TH antisera (Fig. S3, and S4). Thus, these DDC neurons correspond to the presumptive dopaminergic vmTH neurons (see above). The somata of the fourth and the fifth distinct DDC neuron groups reside dorso-laterally at the height of the DL tracts. The fourth group of DDC neurons showed strong DDC and TH immunolabeling. These DDC neurons, therefore, likely represent the presumptive dopaminergic dlTH neurons (Fig. S4; see above), but were usually missing in the *Ddc-gal4* expression pattern. The remaining neurons constitute the fifth distinct DDC neuron group (VL1 neurons; [19]; see also below) since they neither seem to synthesize 5-HT nor DA (Fig. S4). VL1 neurons showed prominent *Ddc-gal4*-driven mCD8GFP expression, but comparably weak DDC immunoreactivity (Fig. S3). The VL1 neuron somata are typically arranged as segmentally reiterated pairs in t3, a1-4, and a6-7, and reside laterally between the height of the VL and the DL tracts. In t3 and a1-4, VL1 neurites initially run ventrally towards the VL tracts, then project beneath the TP 4 ventro-medially until they converge in proximity to the VM tracts. After forming extensive arborizations between the DM and VM tracts, the VL1 neurites proceed dorsally, diverge within the cortex, and project via the segmental nerves to the periphery.

### Tyrosine decarboxylase 2 and tyramine β-hydroxylase neurons

**General characteristics.** Tyrosine decarboxylase (TDC) catalyzes the synthesis of tyramine (TA) from tyrosine. A second enzyme, tyramine β-hydroxylase (TβH), may then convert TA to its metabolite octopamine (OA; see [1], [3], [4], [57]–[59]). In adult *Drosophila*, two different TDC genes, *Tdc1* (CG30445) and *Tdc2* (CG30446), show a largely non-overlapping expression pattern: TDC1 is most abundant in non-neural tissue of the abdomen, but also localizes to a few thoracic neurons of the VG [60]. In contrast, TDC2 is primarily expressed within the CNS [60]. Like *Tdc2*, the *Drosophila Tβh* gene (CG1543) encodes a single protein that is primarily expressed within CNS neurons. According to the enzymatic function of TβH, TβH-immunoreactive neurons show a similar distribution pattern as OA-immunoreactive neurons in the VG [61]. In *Drosophila*, OA has a multitude of modulatory and hormonal functions (see [1], [3], [4], [57]–[59]). For example, OA regulates ovulation and egg laying [60], [62]–[64]. Both OA and TA appear to modulate locomotor activity in the larva [65], [66] and in the adult fly [67], [68]. TA signaling also seems to influence diuresis [69]–[71] and olfactory behavior [72].

Previous morphological descriptions of tyraminergic/octopaminergic neurons in the larval VG relied on immunostainings against TA [73], OA [74], and TβH [61]. In addition, Landgraf et al. [19] mapped the *MzVum-gal4-*expressing neuron group-which appears to include efferent TA-/OA-immunoreactive neurons-in the Fas2 landmark system. We here used a *Tdc2-gal4* line [60] and a polyclonal rat-anti-TβH serum [61] to reveal presumed tyraminergic/octopaminergic neurons (TDC2 neurons) in the VG. Since all TDC2 neurons showed TβH immunoreactivity (Fig. S5), they likely all produce OA. This opposes the recent assumption that some ventral midline neurons may synthesize only TA and not OA [73]. In general, the distribution of TDC2 neurons resembled the TA immunostaining pattern. We observed additional pairs of paramedial TDC2 neurons and one or two dorsal unpaired median TDC2 neuron(s) that could not be detected with the TA antiserum [73]. The TA antiserum instead labeled dorso-laterally located neurons (lTA neurons) in a1-7 [73], which appeared to be missing in the *Tdc2-gal4*-driven mCD8GFP expression pattern. Since the lTA neurons also lacked both OA [74] and TβH immunoreactivity (Fig. S5), they possibly synthesize TA, but no OA. Noteworthy, the number and position of lTA neurons highly resembles that of presumptive dopaminergic dlTH neurons (see above). Thus, lTA neurons may represent unique TA neurons that synthesize TA from DA via the dopamine dehydroxylase pathway, but do not produce OA (Fig. 1; see [4]).

**Figure 8:**
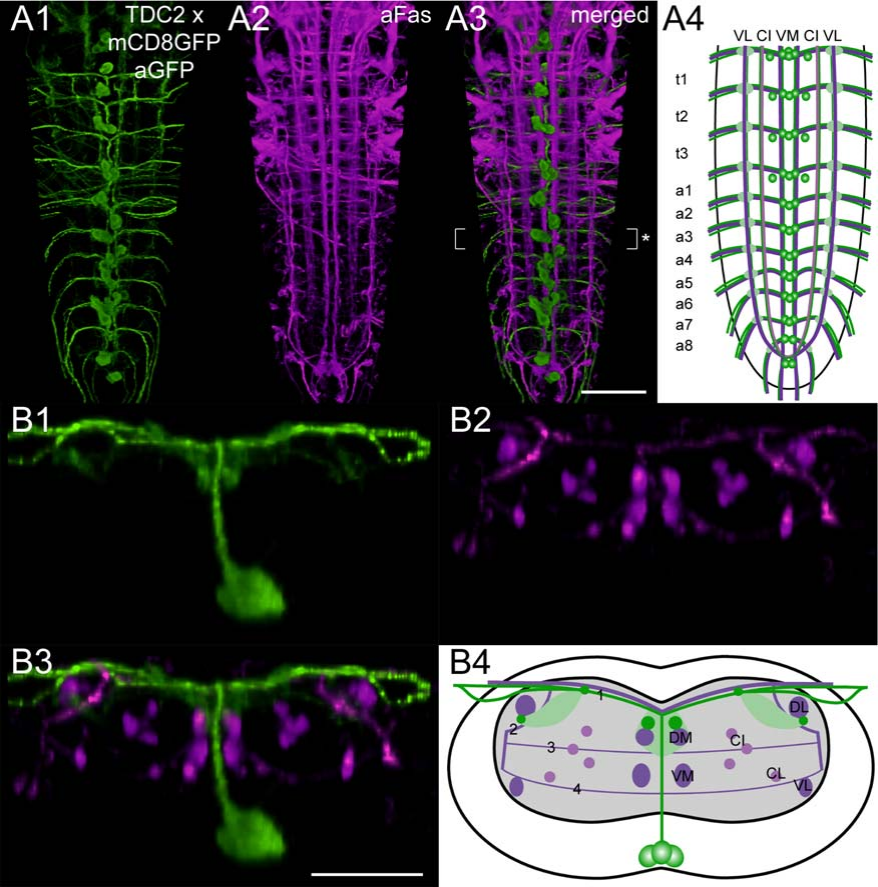
Mapping of *Tdc2-gal4* x mCD8GFP expressing neurons. A1) Ventral view of 3D image stacks showing volume-rendered GFP-immunoreactive neurons (green) and A2) Fas2-positive tracts (magenta) in a larva expressing *Tdc2-gal4*-driven mCD8GFP. The merged image (A3) served for an idealized schematic representation of *Tdc2-gal4* x mCD8GFP expressing neurons in the Fas2 landmark system (A4). B1-3) Corresponding transversal views of the neuromere a3 (* in A3), and B4) the deduced idealized scheme. Note that the idealized scheme A4) includes the pmTDC2 neurons of t1-3 and a1, which often faintly expressed *Tdc2-gal4*-driven mCD8GFP. The pmTDC2 neurons, however, always showed strong TβH immunoreactivity (see Fig. S5). Green-shaded areas in the schemes contain a high concentration of GFP-immunoreactive arborizations. Scale bars: 50 µm in A), 25 µm in B).

**Morphology** (Fig. 8). With respect to their position in the Fas2 landmark system, three distinct TDC2 neuron groups reside in the larval VG: The first group comprises ventral unpaired median TDC2 neurons (vumTDC2 neurons) that locate to the cortex beneath the VM tracts. In general, three vumTDC2 neurons compose a neuron cluster in the neuromeres t1-a6, whereas a7 appears to contain only two vumTDC2 neurons. The second group typically consists of two dorso-medial TDC2 neurons (dmTDC2 neuron) residing between the last subesophageal neuromere and t1, and two dmTDC2 neurons of a8. All dmTDC2 neurons localize to the dorsal cortex above the DM tracts. The third distinct TDC2 neuron group in the VG comprises paramedial neuron pairs (pmTDC2 neurons). The pmTDC2 neuron somata lay in the ventral cortex of t1-3 and a1, somewhat ventro-laterally to the VM fascicles. Typically, pmTDC2 neurons showed faint *Tdc2-gal4*-driven mCD8GFP expression, but strong TβH immunoreactivity (Fig. S5). Prominent longitudinal TDC2 neurites project above the DM tracts. Finer longitudinal projections run at the dorsal neuropil rim above the CI fascicles and along the DL tracts. All of these longitudinal neurites appear to originate from descending TDC2 neurons. The descending TDC2 neurites proceed through the whole length of the VG until they coincide at the tip of the VG. In contrast to the descending TDC2 neurons, vumTDC2 neurons project dorsally, where their neurites form extensive arborizations in the neuropil above the DM tracts. The vumTDC2 neurites then diverge in the dorsal cortex and project above TP 1 laterally towards the longitudinal TDC2 neurites at the CI tracts. There, the vumTDC2 neurites may bifurcate again and traverse above or beneath the DL fascicles. Outside of the VG, vumTDC2 neurites join and project together via the segmental nerves to the periphery. We could not trace the projections of the dm- and pmTDC2 neurons since these neurites lacked *Tdc2-gal4*-driven mCD8GFP expression as well as TβH immunoreactivity (Fig. 8, Fig. S5).

**Figure 9:**
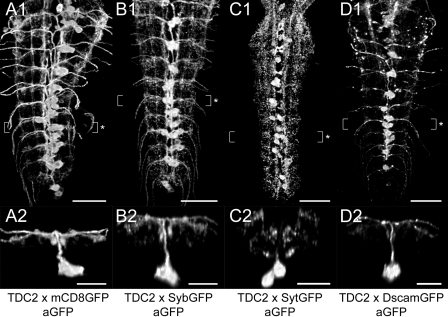
Distribution of ectopically expressed neuronal compartment markers in TDC2 neurons. (A1-D1) Dorsal view of GFP immunoreactivity in the VG of larvae expressing *Tdc2-gal4-*driven mCD8GFP (A1-2), SybGFP (B1-2), SytGFP (C1-2), or DscamGFP (D1-2). (A2-D2) Corresponding transversal views of the neuromeres a3 or a4 (* in A1-D1). All images are auto-contrasted maximum pixel intensity projections of volume-rendered 3D image stacks. Scale bars: 50 µm in A1-D1), 25 µm in A2-D2).

**Distribution of ectopically expressed markers** (Fig. 9). Because *Tdc2-gal4* specifically drove mCD8GFP expression in presumed tyraminergic/octopaminergic neurons, we used the neuronal compartment markers SybGFP, SytGFP and DscamGFP to reveal their putative in- and output zones. The distribution of *Tdc2-gal4*-driven SybGFP highly resembled the mCD8GFP expression pattern. SybGFP localized to the neurites of vumTDC2 neurons and to all longitudinal projections of TDC2 neurons. Furthermore, SybGFP accumulated around peripherally projecting vumTDC2 neurites in the dorso-lateral neuropil between the CI and DL tracts. Compared to SybGFP, *Tdc2-gal4*-driven SytGFP showed a different distribution pattern. SytGFP accumulated around the DM/VM tracts and in the dorso-lateral neuropil. The peripheral projections of the vumTDC2 neurons completely lacked SytGFP. Thus, the SytGFP labeling in the VG appears to belong to descending TDC2 neurons. In contrast to SybGFP and SytGFP, DscamGFP almost exclusively localized to vumTDC2 neuron somata and neurites. Only TDC2 neurites in t1-3 and longitudinal TDC2 projections along the DM and DL tracts also showed a dotted DscamGFP labeling pattern.

### Fas2-based identification of co-localized signaling molecules

**Figure 10:**
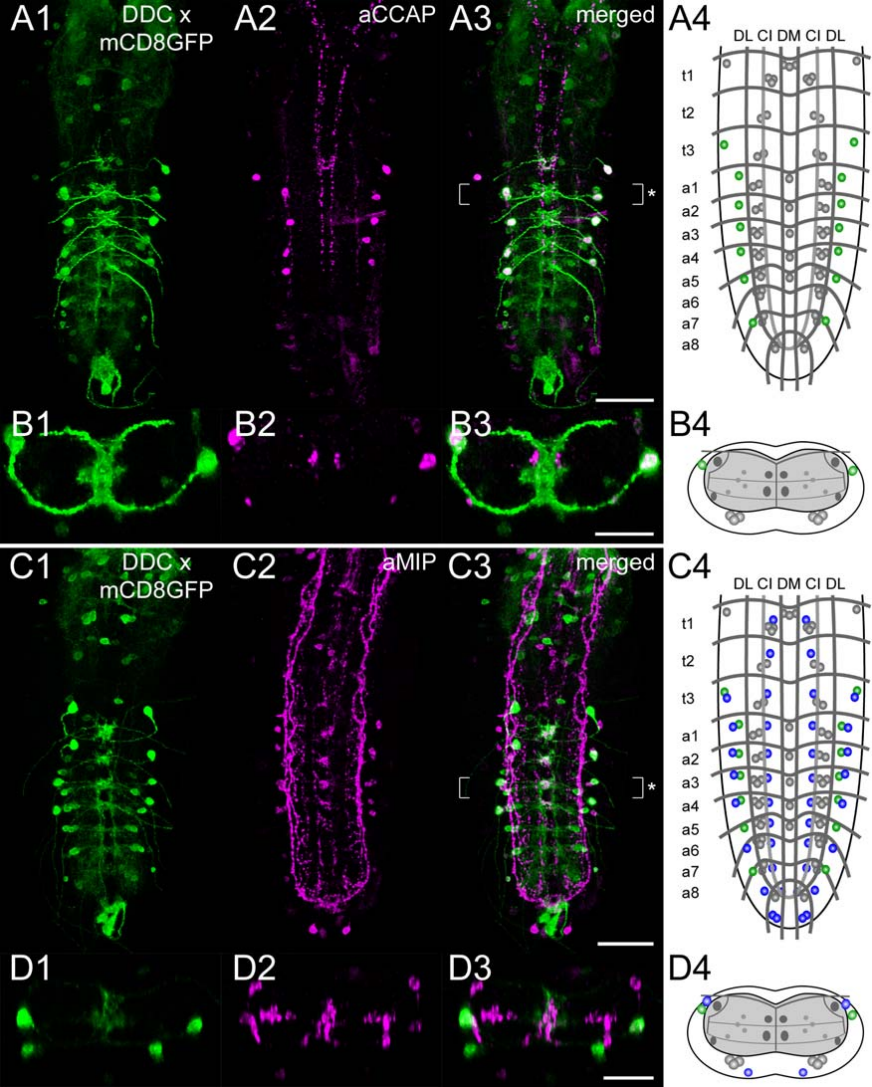
Fas2-based comparison of *Ddc-gal4*-driven mCD8GFP expression and CCAP/MIP immunoreactivity in the larval VG. A1) Dorsal view of *Ddc-gal4* x mCD8GFP expressing neurons (green) and A2) CCAP-immunoreactive neurons (magenta) in a larval VG. The merged image (A3) served for an idealized schematic representation of the respective neuron groups in the Fas2 landmark system (A4). B1-3) Corresponding transversal views of the neuromere a1 (* in A3), and B4) the deduced idealized scheme. C1) Dorsal view of *Ddc-gal4* x mCD8GFP expressing neurons (green) and C2) MIP-immunoreactive neurons (magenta) in a larval VG. The merged image (C3) provided the basis for the scheme shown in (C4). D1-3) Corresponding transversal views of the neuromere a3 (* in C3), and D4) the deduced idealized scheme. In all schemes, neurons showing both *Ddc-gal4*-driven mCD8GFP expression and CCAP/MIP immunoreactivity are green-colored. *Ddc-gal4* x mCD8GFP expressing neurons which lacked CCAP/MIP immunoreactivity are shown in gray. Vice versa, neurons which exclusively showed CCAP/MIP immunoreactivity are blue-colored. Original images are maximum pixel intensity projections of volume-rendered 3D image stacks. Scale bars: 50 µm in A) and C), 25 µm in B) and D).

During our morphological characterization of aminergic neurons in the larval VG, we observed two distinct neuron groups within the *Ddc-gal4*-driven mCD8GFP expression pattern that apparently synthesize neither 5-HT nor DA (Fig. S4; see above). While the ventro-medially residing DDC neuron group obviously synthesizes the neuropeptide corazonin [19], the signaling molecules of the other group, the VL1 neurons, have remained unidentified. We hence applied Fas2-based mapping and compared the relative distribution of VL1 neurons in the VG with that of recently mapped peptidergic neurons [20]. Interestingly, the relative position of VL1 neurons in the Fas2 landmark system highly resembled efferent neurons showing crustacean cardioactive peptide (CCAP) and myoinhibiting peptide (MIP; [20]) immunoreactivity. We employed the respective antisera and confirmed that VL1 neurons indeed are CCAP- and MIP-immunoreactive (Fig. 10). Since *Ddc-gal4*-driven mCD8GFP was generally missing in dlTH neurons, and dlTH neurons showed a similar position within the Fas2 landmark system as CCAP/MIP-synthesizing neurons (CCAP/MIP neurons), we finally tested whether any CCAP/MIP neuron co-expresses TH. However, *Ccap-gal4*-driven mCD8GFP expression and TH immunoreactivity did not overlap, but localized to different neuron groups (Fig. S6).

## Discussion

We here used *gal4*-driven marker gene expression and immunocytochemistry to three-dimensionally map presumed serotonergic, dopaminergic and tyraminergic/octopaminergic neurons within the Fas2 landmark system of the larval VG. Furthermore, we employed several ectopically expressed pre- and postsynaptic markers to reveal the in- and output compartments of presumptive dopaminergic TH and tyraminergic/octopaminergic TDC2 neurons. Our results now allow to compare the segmental distribution patterns of aminergic neurons and to trace aminergic projections to defined neuropil areas within the VG. In the following, we relate the morphology of aminergic neurons to known BA functions and describe putative neuronal network interactions with other VG neurons. We also exemplify how Fas2-based mapping can simplify the identification of co-localized signaling molecules, and allocate all neurons within the complex *Ddc-gal4* expression pattern to distinct neuron subsets.

### Both interneurons and efferent neurons of the larval VG contain BAs

Throughout the insects, similar neuron groups synthesize BAs. These groups typically comprise only few neurons with large branching patterns (see [1]–[3]). In agreement with previous studies [29]–[31], 5-HT neurons in t1-a8 of the *Drosophila* larval VG represent interneurons with intrasegmental neurites. The 5-HT neurons of a8, however, appear to supply only the neuropil of a7, but not that of a8 and the adjacent “terminal plexus”. Like 5-HT neurons, the presumptive dopaminergic TH neurons lack peripheral projections and appear to exclusively represent interneurons, as previously proposed [39]. In contrast, presumptive tyraminergic/octopaminergic TDC2 neurons mostly represent efferent vumTDC2 neurons. The vumTDC2 neurons obviously project to larval body wall muscles including M1 and M2 since these muscles showed TA- [73] and OA-immunoreactive type II boutons [74]. In a8, dorsally located dmTDC2 neurons send axons through the associated segmental nerves, and hence are efferent neurons as well. These dmTDC2 neurons probably innervate the reproductive tract in the adult female fly [63]. Besides the dmTDC2 neurons of a8, typically two additional dmTDC2 neurons reside in the dorsal cortex between the last subesophageal neuromere and t1. These dmTDC2 neurons were not described in previous morphological studies on TA- [73] and OA-/TβH-immunoreactive neurons [61], [74]. Nevertheless, all dmTDC2 neurons in the VG consistently showed strong *Tdc2-gal4*-driven mCD8GFP expression as well as TßH immunoreactivity. Thus, they likely synthesize both TA and OA. Although we could not trace their neurites, the dmTDC2 neurons resemble a pair of anterior medial neurons in locusts [75] and crickets [76] that localize to t1 and innervate the anterior connectives (see [3]). Alternatively, dmTDC2 neurons may correspond to a single dorsal unpaired median neuron which resides in t1 of the locust and supplies the subesophageal nerves [77]. Like dmTDC2 neurons, pmTDC2 neurons are probably interneurons as well. The soma position of pmTDC2 neurons highly resembles that of descending OA-immunoreactive interneurons detected in the subesophageal and thoracic neuromeres of bees, crickets, cockroaches, locusts, and moths (see [3], [78]).

### Aminergic innervation varies between specific VG neuromeres

Within the larval VG of *Drosophila*, aminergic neurons typically show a segmentally reiterated distribution (see [1], [2]). The number of aminergic modules, however, often varies between different neuromeres. 5-HT neurons, for instance, typically occur as two bilateral pairs per neuromere. Yet, t1 comprises three 5-HT neuron pairs and a8 only one pair. The presumptive dopaminergic TH neurons also lack a strict serial homology since three vmTH neurons are present in t1, but only one in t2-a7. Furthermore, dlTH neurons locate to a1-7, but appear to be missing in t1-3. The neuromere a8 lacks TH neurons. The number of presumptive tyraminergic/octopaminergic TDC2 neurons differs between various neuromeres as well. Whereas t1 comprises one or two dmTDC2 neurons, comparable neurons are absent in t2-a7. Putative descending pmTDC2 interneurons localize to t1-a1, but appear to be missing in the remaining abdominal neuromeres. Taken together, the number of aminergic modules in t1 and a8 often deviated from that of t2-a7. This difference may-at least partially-reflect unique neuronal circuits in t1 and a8. While we are not aware of t1 specific physiological functions in larvae, a8 and the adjacent “terminal plexus” are associated with the tail region, and hence contain a specific set of sensory neurons and motoneurons. The terminal neuromeres also supply several unique structures such as the spiracles or the anal pads (see [79], [80].

Besides the segmental differences in neuron number, the density of aminergic innervation and the amount of immunolabeling/marker gene expression varies between neuromeres as well. In particular, presumptive dopaminergic TH neurons show a striking neuromere-specific labeling pattern. Whereas a1-5 contain only few labeled TH projections, t1-3 and a6-7 comprise a comparably dense network of TH neurites. Similar to TH neurons, 5-HT neurons most densely innervate the neuropil of a7. Since a high extracellular concentration of 5-HT decreases the density of 5-HT-immunoreactive arborizations within the neuropil [81], a7 may represent a minor 5-HT release site. In contrast to a7, the neuropil of a8 and the adjacent “terminal plexus” (- which receive prominent peptidergic innervation [20] -) typically lack aminergic neurite arborizations. Consequently, larval aminergic neurons may play a subordinate role in tail-related physiological processes.

### Ectopically expressed fluorescent fusion proteins as neuronal compartment markers in aminergic neurons

To reveal putative synaptic in- and output zones of aminergic neurons, we employed the neuronal compartment markers neuronal synaptobrevin-GFP, synaptotagmin 1-GFP, and *Drosophila* Down syndrome adhesion molecule [17.1]-GFP. Neuronal synaptobrevin is a vesicle associated membrane protein that plays a role in the SNARE complex during vesicle transport and fusion with the plasma membrane (see [82]). In accordance with this function, ectopically expressed neuronal synaptobrevin-GFP (SybGFP) accumulates at nerve terminals [50], [51]. SybGFP therefore served to define the presynaptic compartments of several *Drosophila* neurons, e.g. in the visual system [83]–[85]. However, neuronal synaptobrevin is not restricted to small synaptic vesicles, but also locates to the membrane of large dense core vesicles, which contain BAs or neuropeptides (see [86]–[89]). Consequently, in a7, SybGFP localized to putative release sites of presumptive serotonergic DDC neurons [81]. SybGFP was also used to identify non-synaptic release sites in several peptidergic neurons [20], [90]. In aminergic neurons, the distribution of *gal4*-driven SybGFP highly resembled the corresponding mCD8GFP expression pattern. SybGFP localized in dotted patterns to aminergic neuron somata and associated neurites. We therefore suggest that SybGFP does not exclusively label the presynaptic compartments of aminergic neurons. This fits to the assumption that ectopically expressed synaptic proteins can either localize to transport vesicles or non-synaptic compartments in peptidergic neurons [91]. On the other hand, the ubiquitous distribution of SybGFP in aminergic neurites may suggest a widespread BA release/recycling from non-synaptic active sites. In mammals, BA release/recycling is not restricted to synapses [92], [93]. Vesicular monoamine transporters, which transport BAs into secretory vesicles, reside within neuron somata, axons, and dendrites [92]. In *Drosophila*, the vesicular monoamine transporter DVMAT-A localizes to somata as well as neurites of several aminergic neurons both in the larval [94] and adult CNS [38]. Thus, the widespread distribution of SybGFP and DVMAT-A in aminergic neurons suggests that a considerable amount of aminergic vesicles resides at non-synaptic sites. Non-synaptic BA release/recycling might therefore play a major role for aminergic neuronal network signaling.

Like neuronal synaptobrevin, synaptotagmins also represent integral membrane proteins of both small synaptic and large dense core vesicles (see [86]–[89]). In *Drosophila*, the products of seven synaptotagmin genes localize to distinct neuronal compartments including the postsynaptic site [95]. At the presynaptic site, synaptotagmin 1 does not participate in the SNARE complex, but acts as a Ca^2+^-sensor for synaptic vesicle fusion (see [96]). Furthermore, synaptotagmin 1 appears to be the only crucial isoform for synaptic vesicle release [97]–[99]. Consequently, a synaptotagmin 1-GFP fusion construct (SytGFP) was developed as a synaptic vesicle marker that specifically labels presynaptic sites [52]. In aminergic neurons, the distribution pattern of SytGFP strikingly differed from the observed mCD8GFP and SybGFP labeling. Primary neurites of aminergic neurons always completely lacked SytGFP. Varicose neurite structures which were less evident in the mCD8GFP and SybGFP expression patterns showed strong SytGFP labeling. In agreement with the SytGFP distribution in other *Drosophila* neuron types [52], [100], [101], SytGFP hence appears to exclusively accumulate at the presynaptic sites of aminergic neurons. Thus, SytGFP represents a valuable marker to separate synapses from other neuronal compartments in aminergic neurons. However, since BA release is not restricted to synapses, SytGFP may not label all BA release sites of aminergic neurons. The sparse co-localization of SytGFP and SybGFP in aminergic neurites in fact suggests that aminergic vesicles-which are located distal to presynaptic sites-generally lack SytGFP. Consequently, non-synaptic BA release appears to be independent of synaptotagmin 1, but may depend on other synaptotagmin isoforms such as synaptotagmin α or β [95]. The differing distribution of SytGFP and SybGFP also suggests that aminergic neurons contain several types of aminergic vesicles which are either associated with presynaptic or non-synaptic BA release. Alternatively, aminergic neurons may synthesize additional non-aminergic neurotransmitters like acetylcholine, GABA, or glutamate. Presumed octopaminergic efferent neurons, for instance, appear to release glutamate from type II terminals at the neuromuscular junction [94]. In such neurons, SytGFP likely labels presynaptically located transmitter vesicles and may not reveal BA release sites.

In contrast to SybGFP and SytGFP, ectopically expressed *Drosophila* Down syndrome adhesion molecule [17.1]-GFP (DscamGFP) localized to postsynaptic compartments and not to axons or presynaptic sites [53]. Consequently, DscamGFP recently served as dendrite marker in mushroom body lobe neurons [101] and peptidergic neurons [102]. Aminergic neurons showed only weak DscamGFP labeling. DscamGFP primarily localized to neurites that lacked SytGFP labeling. Since SytGFP accumulates at presynaptic sites, DscamGFP appears to represent a valuable marker to define dendritic compartments in aminergic neurons.

### Neuronal compartments of 5-HT neurons

In 5-HT neurons, we did not analyze the distribution of ectopically expressed neuronal compartment markers since specific *gal4* drivers are not available. The *Ddc-gal4* driver induces marker gene expression not only in presumed serotonergic, but also in dopaminergic and additional peptidergic neurons (see below). Consequently, neurites of different DDC neuron subsets overlap in specific neuropil areas. Presumptive serotonergic as well as dopaminergic DDC neurites, for instance, localize to the VG neuropil above the CI tracts. These conditions prevent an accurate description and interpretation of the compartment marker distribution in presumptive serotonergic DDC neurons. Thus, appropriate *gal4* drivers (e.g. *Dtph-gal4*) are needed to further analyze 5-HT neuron morphology.

### Possible overlap of 5-HT neurons with other neuronal projections

5-HT neurons bifurcate strongly in the whole neuropil of t1-a7, and hence may influence various VG neurons including sensory, inter- as well as motoneurons. However, we did not analyze putative neuronal network contacts of 5-HT neurons since previous morphological studies on *Drosophila* 5-HT receptors [12], [13] did not describe the exact spatial location of the respective receptors in the larval VG.

### Neuronal compartments of dopaminergic TH neurons

In TH neurons, the distribution of ectopically expressed mCD8GFP, SybGFP, SytGFP and DscamGFP differed only slightly. This might relate to the fact that the VG contains two different TH neuron groups, the vmTH and dlTH neurons, whose neurites contact each other at longitudinal projections. Consequently, pre- and postsynaptic compartments of both TH neuron groups appeared to overlap, e.g. at longitudinal projections next to the VL tracts. Since additional TH neurons located in the brain or subesophageal ganglia also innervate the VG [1], we could not clarify which TH neuron group attributes to a particular neuronal projection. Several morphological findings, however, suggest that TH neurons possess distinct in- and output sites: Most strikingly, a1-5 contained less TH neurites labeled with mCD8GFP, SybGFP and DscamGFP, as compared to t1-3 and a6-7. In t1-a7, high amounts of SybGFP and SytGFP located to lateral longitudinal projections next to the VL tracts. These longitudinal TH neurites also contained a comparably high amount of DscamGFP, and hence likely represent synaptic in- as well as output compartments of different TH neuron groups. Besides lateral longitudinal TH projections, SybGFP and SytGFP also co-localized to the median neuropil between the DM/VM tracts. At least in a1-5, this neuropil area lacked DscamGFP, and hence probably represents a presynaptic output site of TH neurons. In a6-7, we observed a comparably strong SybGFP and SytGFP labeling in arborizations around transversal TH neurites. Whereas SybGFP mainly located to the dorsal branches of the transversal TH neurite loops, SytGFP and DscamGFP primarily labeled the ventral branches. Thus, the dorsal branches of the transversal TH neurite loops may represent non-synaptic DA release sites, while the ventral branches seem to comprise overlapping synaptic in- and output compartments of different TH neuron groups.

### Possible overlap of TH neurites with other neuronal projections

Both vmTH and dlTH neurons innervate distinct neuropil areas within the VG. The vmTH neurons send their primary neurites dorsally and then project through the dorsal part of the neuropil above TP 3. Since the dorsal neuropil comprises the dendritic compartments of most motoneurons [19], vmTH neurites are ideally located to modulate locomotor activity. This fits to the finding that DA application onto intact larval CNS-segmental preparations rapidly decreased the rhythmicity of CNS motor activity and synaptic vesicle release at the neuromuscular junction [103]. Unlike vmTH neurons, dlTH neurons exclusively innervate the ventral part of the VG neuropil beneath TP 3. There, putative dendritic compartments of TH neurons mainly localize to lateral longitudinal and to transversal projections adjacent to the main output site of several afferent sensory neurons, e.g. tactile and proprioreceptive neurons [19], [21], [104]. Thus, some TH neurons may receive synaptic input from specific sensory neurons. On the other hand, TH neurons also seem to have output sites in the ventral part of the neuropil, and hence may influence the signal transmission between sensory neurons and interneurons. This fits to the finding that peptidergic *apterous* neurons, which appear to transmit sensory input from the VG to the brain [105], express DA receptors [8], [106]. Concomitantly, dendritic compartments of *apterous* neurons [91] seem to reside adjacent to the putative DA release sites of TH neurons at the CI tracts. Besides the overlap between transversal TH neurites and sensory/interneuron projections in the ventral neuropil, TH neurons may influence several neuron groups at other locations within the VG. For instance, the putative synaptic output sites of TH neurons in the median neuropil between the DM/VM tracts overlap with presumptive input compartments of both interneurons and efferent neurons expressing peptides such as CCAP, corazonin, FMRFa, or MIP [20]. Furthermore, the putative output sites at longitudinal TH projections next to the VL tracts lay adjacent to presumptive input compartments of e.g. efferent leucokininergic neurons [20].

### Neuronal compartments of tyraminergic/octopaminergic TDC2 neurons

In the VG, most TDC2 neurons are efferent vumTDC2 neurons and showed a differential distribution of ectopically expressed SybGFP, SytGFP, and DscamGFP. The primary neurites and transversal projections of vumTDC2 neurons were labeled with DscamGFP, but lacked SytGFP. Therefore, these neurites likely represent dendritic input sites. This fits to the finding that vumTDC2 neurons possess output sites at larval body wall muscles [73], [74]. However, vumTDC2 neurites within the VG also contained high amounts of SybGFP, and hence may release TA/OA from non-synaptic sites. Besides vumTDC2 neurites, SybGFP strongly labeled longitudinal TDC2 neurites and associated arborizations in the dorso-lateral neuropil between TP 1 and 3. These TDC2 projections showed prominent SytGFP labeling and TßH immunoreactivity, but largely lacked DscamGFP. Thus, the dorsal part of the VG neuropil likely contains output compartments of TDC2 neurons. Since the larval brain seems to contain only tyramine- [73] and no octopamine-immunoreactive neurons [1], [61], these output sites likely derive from descending interneurons located in the subesophageal ganglia, dmTDC2 or pmTDC2 neurons. Noteworthy, the strong SybGFP and SytGFP labeling in TDC2 neurites projecting through the dorso-lateral neuropil of the VG overlapped with DscamGFP in transverse vumTDC2 neurites. Thus, descending TDC2 neurons may interact with vumTDC2 neurons.

### Possible overlap of TDC2 neurons with other neuronal projections

The VG comprises efferent vumTDC2 neurons as well as several putative TDC2 interneuron groups. Since all vumTDC2 neurons appear to have synapses at peripheral targets [73], [74] and dendrites in the dorsal neuropil, they show the typical motoneuron morphology. This corresponds to the finding that OA inhibited synaptic transmission at the neuromuscular junction by affecting both pre- and postsynaptic mechanisms [107]. In addition, TβH mutant larvae, with altered levels of TA and OA, showed severe locomotion defects [66], which seemed to be linked to an imbalance between TA and OA signaling [65]. Hence, vumTDC2 neurons likely regulate peripheral processes such as body wall muscle activity, whereas TDC2 interneurons centrally modulate the neuronal activity of motoneurons and interneurons involved in locomotor control. Interestingly, presumptive presynaptic compartments of descending TDC2 interneurons reside adjacent to transversal vumTDC2 dendrites. Thus, both TDC2 neuron groups may interact to modulate larval locomotor activity. Besides their function for locomotion, descending TDC2 neurons may also influence other neurons which project into the dorsal neuropil between TP 1 and 3. The putative output sites of TDC2 interneurons, for instance, lay adjacent to several peptidergic projections showing allatostatin-A, FMRFa, MIP or tachykinin immunoreactivity [20]. However, nothing is known about TA/OA receptor distribution in the larval VG.

### Fas2-based fractionation and definition of the complex *Ddc-gal4* expression pattern reveals *Ddc* expression in peptidergic neurons

During our morphological analysis of DDC neurons in the L3 larval VG, we identified two DDC neuron groups that obviously synthesize neither 5-HT nor DA. This corresponds to the previous finding that *Ddc-gal4*-driven marker gene expression is not restricted to presumptive serotonergic 5-HT and dopaminergic TH neurons [19]. However, we can not exclude that the putative non-aminergic DDC neurons transiently synthesize BAs during other developmental stages. In our preparations, *Ddc-gal4*-driven mCD8GFP expression never revealed the dlTH neurons. This may relate to the fact that the onset of *Ddc* expression varies between different DDC neuron groups, and high DDC and TH levels do not temporally coincide [29]. Taken together, our results suggest that-at least in the L3 larval VG-the *Ddc-gal4* expression pattern 1) contains additional non-aminergic neurons, and 2) typically comprises most, but not all 5-HT and TH neurons. These particular characteristics of the *Ddc-gal4* driver line should be carefully considered for the interpretation of studies that employed *Ddc-gal4*-driven expression to genetically manipulate serotonergic or dopaminergic neurons. Nevertheless, since all *Ddc-gal4* expressing neurons within the VG showed at least faint DDC immunoreactivity, the *Ddc-gal4* driver appears to restrict ectopical gene expression to DDC neurons. Noteworthy, the DDC neurons which lacked 5-HT and TH immunoreactivity showed corazonin and CCAP/MIP immunoreactivity respectively. In the moth *Manduca sexta*, these peptides play vital roles during ecdysis (see [108]). At least the CCAP/MIP neurons are also necessary for the proper timing and execution of ecdysis behavior in *Drosophila* (see [18]). Since dopaminergic DDC neurons regulate the titers of the molting hormones 20-hydroxyecdyson and juvenile hormone [109], both aminergic and peptidergic DDC neurons may interact to control ecdysis-related events. Recent findings indeed suggest that CCAP/MIP neurons modulate TH activity after eclosion to control the precise onset of tanning [110].

## Supporting Information

Figure S1 Comparison of Th-gal4-driven mCD8GFP expression and GFP immunoreactivity in the larval VG. A1) Dorsal view of Th-gal4 x mCD8GFP expressing neurons (green) in a larval VG, and A2) the corresponding GFP immunostaining (magenta). A3) In the merged image, Th-gal4-driven mCD8GFP co-localizes with GFP immunoreactivity. B1-3) Corresponding transversal views of the neuromere t1 (* in A3), and C1-3) the neuromere a1 ({degree sign} in A3). Note that Th-gal4-driven mCD8GFP expression usually stayed below the detection threshold in ventral TH neurons of a1-7. These TH neurons, however, could be visualized with the GFP antiserum. All images are maximum pixel intensity projections of volume-rendered 3D image stacks. Scale bars: 50 µm in A), 25 µm in B) and C).
(1.67 MB TIF)

Figure S2 Comparison of Th-gal4-driven mCD8GFP expression and TH immunoreactivity in the larval VG. A1) Dorsal view of Th-gal4 x mCD8GFP expressing neurons (green), and A2) TH-immunoreactive neurons (magenta) in a larval VG. A3) In the merged image, Th-gal4-driven mCD8GFP largely co-localizes with TH immunoreactivity. A4) Idealized schematic representation of the respective neuron groups in the Fas2 landmark system. B1-3) Corresponding transversal views of the neuromere a2 (* in A3), and B4) the deduced idealized scheme. In the schemes, neurons showing both Th-gal4-driven mCD8GFP expression and TH immunoreactivity are green-colored. A few ventral neurons typically showed very faint or even lacked Th-gal4-driven mCD8GFP expression, but strongly stained with the TH antiserum (white arrow in B3). These neurons are blue-colored in the schemes. Original images are maximum pixel intensity projections of volume-rendered 3D image stacks. Scale bars: 50 µm in A), 25 µm in B).
(1.10 MB TIF)

Figure S3 Comparison of Ddc-gal4-driven mCD8GFP expression and DDC immunoreactivity in the larval VG. A1) Ventral view of Ddc-gal4 x mCD8GFP expressing neurons (green), and A2) DDC-immunoreactive neurons (magenta) in a larval VG. The merged image (A3) served for an idealized schematic representation of the respective neuron groups in the Fas2 landmark system (A4). B1-3) Corresponding transversal views of the neuromere a4 (* in A3), and B4) the deduced idealized scheme. In the schemes, neurons showing both Ddc-gal4-driven mCD8GFP expression and DDC immunoreactivity are green-colored. Ddc-gal4 x mCD8GFP expressing neurons which showed very faint DDC immunoreactivity are shown in gray (white arrows in B3). Vice versa, neurons which appeared to lack Ddc-gal4 x mCD8GFP expression and showed prominent DDC immunoreactivity are blue-colored (gray arrowhead in B3). Original images are maximum pixel intensity projections of volume-rendered 3D image stacks. Scale bars: 50 µm in A), 25 µm in B).
(1.61 MB TIF)

Figure S4 Comparison of Ddc-gal4-driven mCD8GFP expression and TH or 5-HT immunoreactivity in the larval VG. A1) Dorsal view of Ddc-gal4 x mCD8GFP expressing neurons (green), and A2) TH-immunoreactive neurons (magenta) in a larval VG. The merged image (A3) served for an idealized schematic representation of the respective neuron groups in the Fas2 landmark system (A4). B1-3) Corresponding transversal views of the neuromere a4 (* in A3), and B4) the deduced idealized scheme. C1) Dorsal view of Ddc-gal4 x mCD8GFP expressing neurons (green), and C2) 5-HT-immunoreactive neurons (magenta) in a larval VG. The merged image (C3) provided the basis for the scheme shown in (C4). D1-3) Corresponding transversal views of the neuromere a3 (* in C3), and D4) the deduced idealized scheme. Note that one of the three Ddc-gal4 x mCD8GFP expressing neuron pairs which reside in the ventral cortex always lacked 5-HT immunoreactivity (white arrows in D3). In all schemes, neurons showing both Ddc-gal4-driven mCD8GFP expression and TH/5-HT immunoreactivity are green-colored. Ddc-gal4 x mCD8GFP expressing neurons which lacked TH/5-HT immunoreactivity are shown in gray. Vice versa, neurons which exclusively showed TH/5-HT immunoreactivity are blue-colored. Original images are maximum pixel intensity projections of volume-rendered 3D image stacks. Scale bars: 50 µm in A) and C), 25 µm in B) and D).
(2.93 MB TIF)

Figure S5 Comparison of Tdc2-gal4-driven mCD8GFP expression and TμH immunoreactivity in the larval VG. A1) Ventral view of Tdc2-gal4 x mCD8GFP expressing neurons (green), and A2) TβH-immunoreactive neurons (magenta) in a larval VG. A3) In the merged image, Tdc2-gal4-driven mCD8GFP largely co-localizes with TβH immunoreactivity. A4) Idealized schematic representation of the respective neuron groups in the Fas2 landmark system. B1-3) Corresponding transversal views of the neuromere a1 (* in A3), and B4) the deduced idealized scheme. Due to high TβH background staining, the dorsal half of the VG (dotted line in B3) was omitted in the 3D image stack to reveal co-labeling between Tdc2-gal4 x mCD8GFP expressing and TβH-immunoreactive neurons. In the schemes, neurons showing both Tdc2-gal4-driven mCD8GFP expression and TβH immunoreactivity are green-colored. The paramedial neurons in t2-3 and a1 typically showed very faint or even lacked Tdc2-gal4-driven mCD8GFP expression, but strongly stained with the TβH antiserum (white arrows in B3). These neurons are blue-colored in the schemes. Original images are maximum pixel intensity projections of volume-rendered 3D image stacks. Scale bars: 50 µm in A), 25 µm in B).
(1.85 MB TIF)

Figure S6 Comparison of Ccap-gal4-driven mCD8GFP expression and TH immunoreactivity in the larval VG. A1) Dorsal view of Ccap-gal4 x mCD8GFP expressing neurons (green), and A2) TH-immunoreactive neurons (magenta) in a larval VG. A3) In the merged image, Ccap-gal4-driven mCD8GFP expression and TH immunoreactivity localized to different neuron groups. A4) Idealized schematic representation of the respective neuron groups in the Fas2 landmark system. B1-3) Corresponding transversal views of the neuromere a1 (* in A3), and B4) the deduced idealized scheme. In the schemes, all Ccap-gal4 x mCD8GFP expressing neurons lacked TH immunoreactivity and are shown in gray. Neurons which only showed TH immunoreactivity are blue-colored. Original images are maximum pixel intensity projections of volume-rendered 3D image stacks. Scale bars: 50 µm in A), 25 µm in B).
(1.04 MB TIF)
