# Supplementary figures and images for: Neuroarchitecture of Aminergic Systems in the Larval Ventral Ganglion of Drosophila melanogaster
Source: PLoS One. 2008 Mar 26;3(3):e1848. doi: 10.1371/journal.pone.0001848 (PMC2268740; doi:10.1371/journal.pone.0001848)

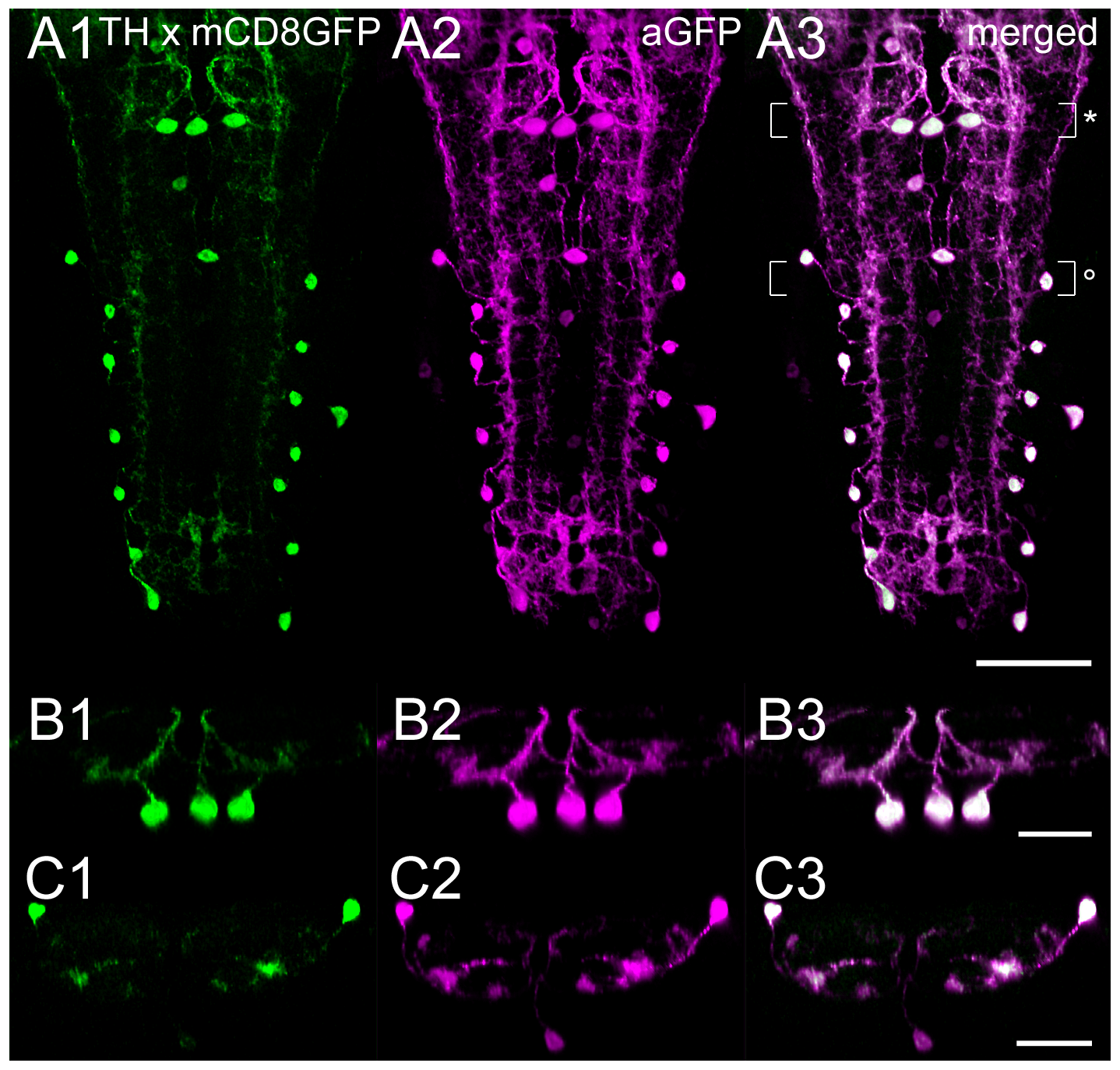

Supplement: Figure S1 — Comparison of Th-gal4-driven mCD8GFP expression and GFP immunoreactivity in the larval VG. A1) Dorsal view of Th-gal4 x mCD8GFP expressing neurons (green) in a larval VG, and A2) the corresponding GFP immunostaining (magenta). A3) In the merged image, Th-gal4-driven mCD8GFP co-localizes with GFP immunoreactivity. B1-3) Corresponding transversal views of the neuromere t1 (* in A3), and C1-3) the neuromere a1 ({degree sign} in A3). Note that Th-gal4-driven mCD8GFP expression usually stayed below the detection threshold in ventral TH neurons of a1-7. These TH neurons, however, could be visualized with the GFP antiserum. All images are maximum pixel intensity projections of volume-rendered 3D image stacks. Scale bars: 50 µm in A), 25 µm in B) and C). (1.67 MB TIF) [file pone.0001848.s001.tif]

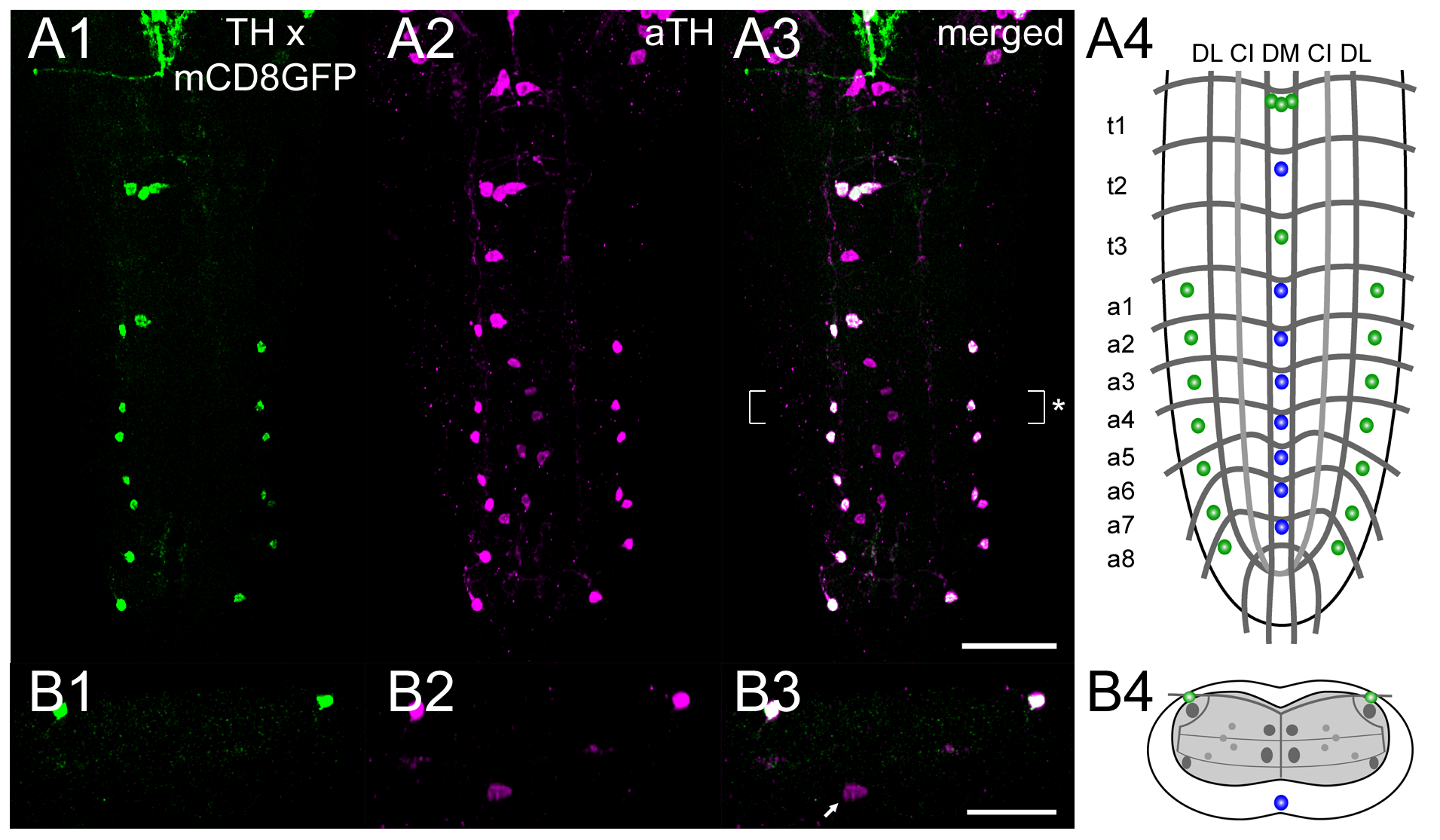

Supplement: Figure S2 — Comparison of Th-gal4-driven mCD8GFP expression and TH immunoreactivity in the larval VG. A1) Dorsal view of Th-gal4 x mCD8GFP expressing neurons (green), and A2) TH-immunoreactive neurons (magenta) in a larval VG. A3) In the merged image, Th-gal4-driven mCD8GFP largely co-localizes with TH immunoreactivity. A4) Idealized schematic representation of the respective neuron groups in the Fas2 landmark system. B1-3) Corresponding transversal views of the neuromere a2 (* in A3), and B4) the deduced idealized scheme. In the schemes, neurons showing both Th-gal4-driven mCD8GFP expression and TH immunoreactivity are green-colored. A few ventral neurons typically showed very faint or even lacked Th-gal4-driven mCD8GFP expression, but strongly stained with the TH antiserum (white arrow in B3). These neurons are blue-colored in the schemes. Original images are maximum pixel intensity projections of volume-rendered 3D image stacks. Scale bars: 50 µm in A), 25 µm in B). (1.10 MB TIF) [file pone.0001848.s002.tif]

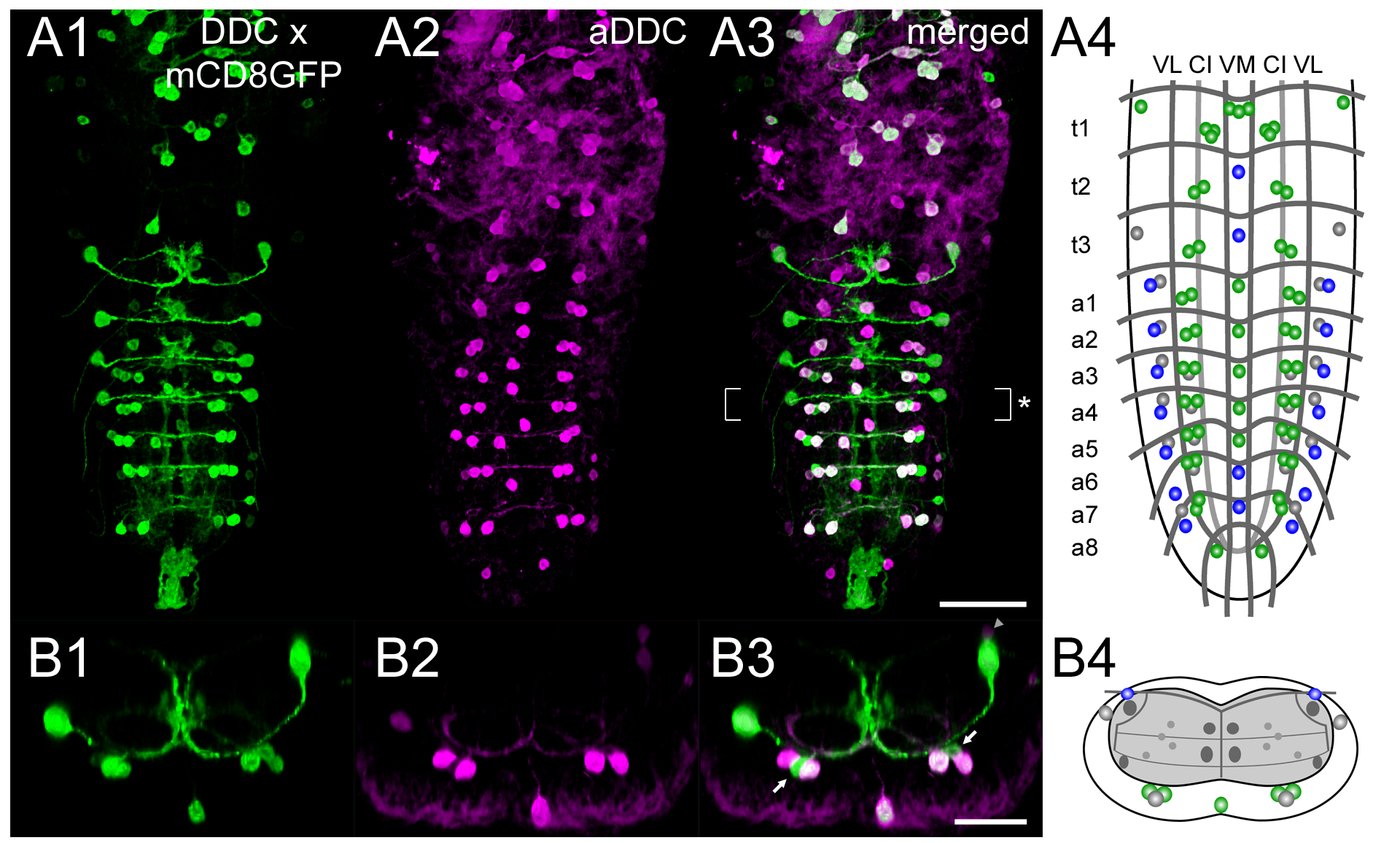

Supplement: Figure S3 — Comparison of Ddc-gal4-driven mCD8GFP expression and DDC immunoreactivity in the larval VG. A1) Ventral view of Ddc-gal4 x mCD8GFP expressing neurons (green), and A2) DDC-immunoreactive neurons (magenta) in a larval VG. The merged image (A3) served for an idealized schematic representation of the respective neuron groups in the Fas2 landmark system (A4). B1-3) Corresponding transversal views of the neuromere a4 (* in A3), and B4) the deduced idealized scheme. In the schemes, neurons showing both Ddc-gal4-driven mCD8GFP expression and DDC immunoreactivity are green-colored. Ddc-gal4 x mCD8GFP expressing neurons which showed very faint DDC immunoreactivity are shown in gray (white arrows in B3). Vice versa, neurons which appeared to lack Ddc-gal4 x mCD8GFP expression and showed prominent DDC immunoreactivity are blue-colored (gray arrowhead in B3). Original images are maximum pixel intensity projections of volume-rendered 3D image stacks. Scale bars: 50 µm in A), 25 µm in B). (1.61 MB TIF) [file pone.0001848.s003.tif]

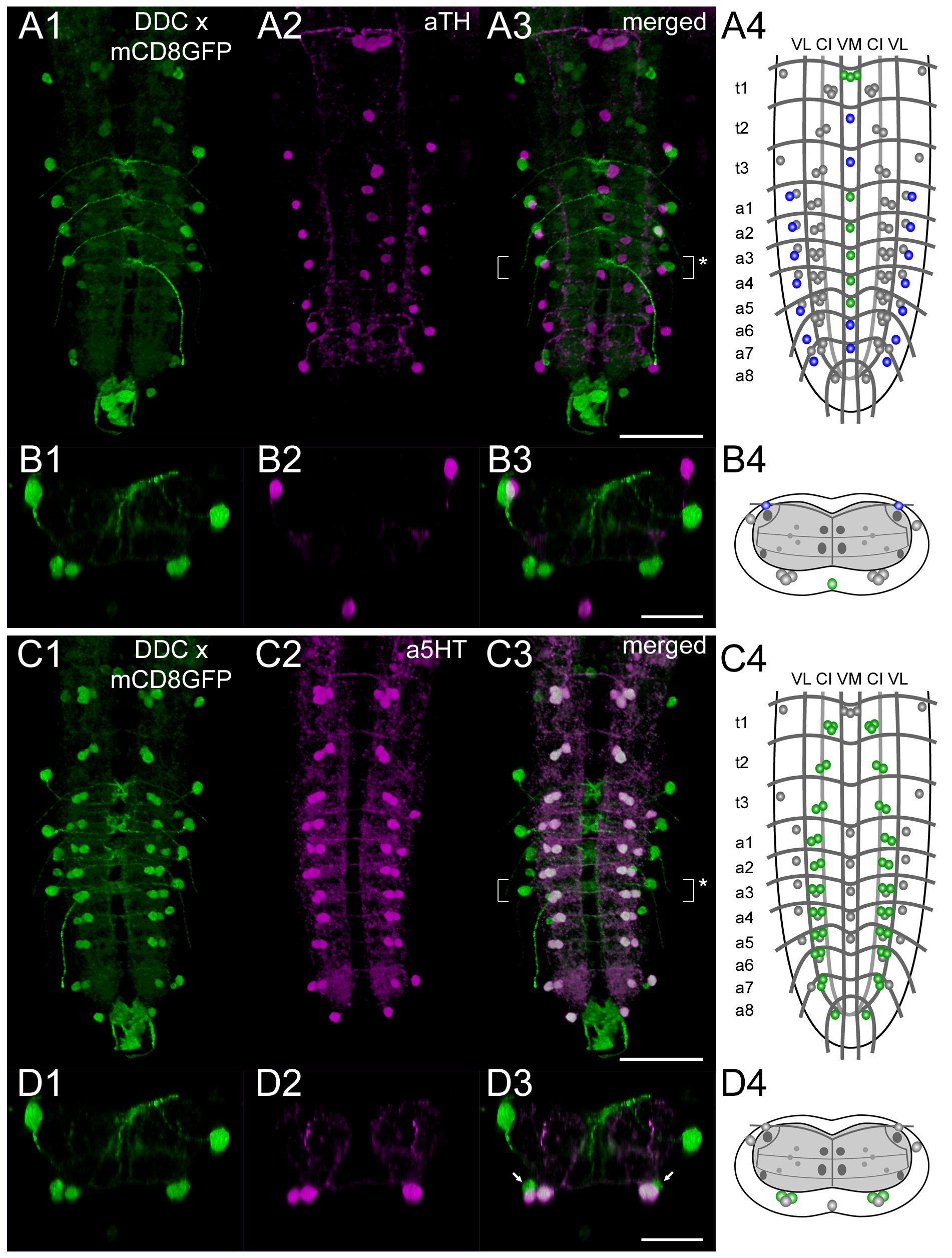

Supplement: Figure S4 — Comparison of Ddc-gal4-driven mCD8GFP expression and TH or 5-HT immunoreactivity in the larval VG. A1) Dorsal view of Ddc-gal4 x mCD8GFP expressing neurons (green), and A2) TH-immunoreactive neurons (magenta) in a larval VG. The merged image (A3) served for an idealized schematic representation of the respective neuron groups in the Fas2 landmark system (A4). B1-3) Corresponding transversal views of the neuromere a4 (* in A3), and B4) the deduced idealized scheme. C1) Dorsal view of Ddc-gal4 x mCD8GFP expressing neurons (green), and C2) 5-HT-immunoreactive neurons (magenta) in a larval VG. The merged image (C3) provided the basis for the scheme shown in (C4). D1-3) Corresponding transversal views of the neuromere a3 (* in C3), and D4) the deduced idealized scheme. Note that one of the three Ddc-gal4 x mCD8GFP expressing neuron pairs which reside in the ventral cortex always lacked 5-HT immunoreactivity (white arrows in D3). In all schemes, neurons showing both Ddc-gal4-driven mCD8GFP expression and TH/5-HT immunoreactivity are green-colored. Ddc-gal4 x mCD8GFP expressing neurons which lacked TH/5-HT immunoreactivity are shown in gray. Vice versa, neurons which exclusively showed TH/5-HT immunoreactivity are blue-colored. Original images are maximum pixel intensity projections of volume-rendered 3D image stacks. Scale bars: 50 µm in A) and C), 25 µm in B) and D). (2.93 MB TIF) [file pone.0001848.s004.tif]

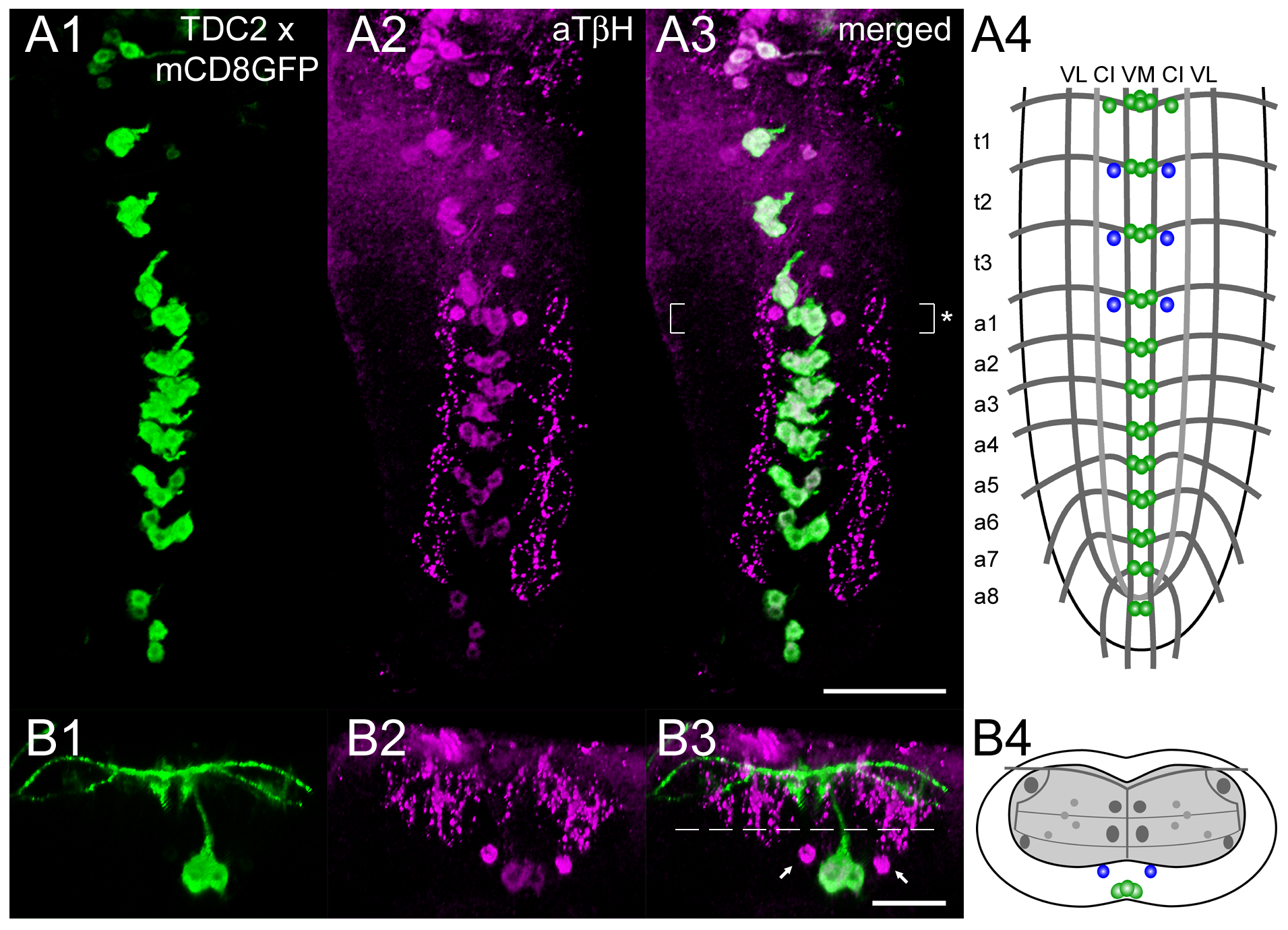

Supplement: Figure S5 — Comparison of Tdc2-gal4-driven mCD8GFP expression and TμH immunoreactivity in the larval VG. A1) Ventral view of Tdc2-gal4 x mCD8GFP expressing neurons (green), and A2) TβH-immunoreactive neurons (magenta) in a larval VG. A3) In the merged image, Tdc2-gal4-driven mCD8GFP largely co-localizes with TβH immunoreactivity. A4) Idealized schematic representation of the respective neuron groups in the Fas2 landmark system. B1-3) Corresponding transversal views of the neuromere a1 (* in A3), and B4) the deduced idealized scheme. Due to high TβH background staining, the dorsal half of the VG (dotted line in B3) was omitted in the 3D image stack to reveal co-labeling between Tdc2-gal4 x mCD8GFP expressing and TβH-immunoreactive neurons. In the schemes, neurons showing both Tdc2-gal4-driven mCD8GFP expression and TβH immunoreactivity are green-colored. The paramedial neurons in t2-3 and a1 typically showed very faint or even lacked Tdc2-gal4-driven mCD8GFP expression, but strongly stained with the TβH antiserum (white arrows in B3). These neurons are blue-colored in the schemes. Original images are maximum pixel intensity projections of volume-rendered 3D image stacks. Scale bars: 50 µm in A), 25 µm in B). (1.85 MB TIF) [file pone.0001848.s005.tif]

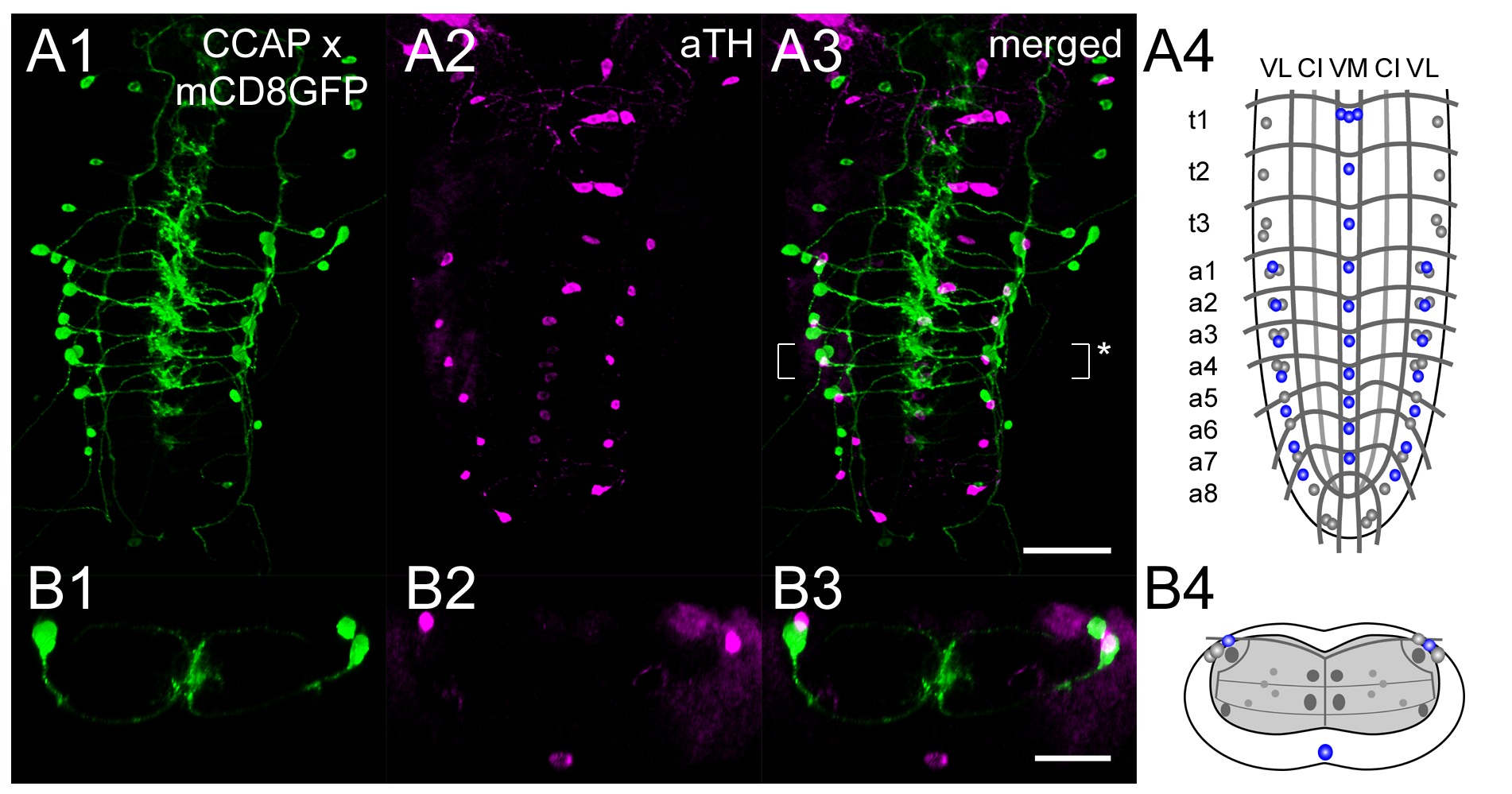

Supplement: Figure S6 — Comparison of Ccap-gal4-driven mCD8GFP expression and TH immunoreactivity in the larval VG. A1) Dorsal view of Ccap-gal4 x mCD8GFP expressing neurons (green), and A2) TH-immunoreactive neurons (magenta) in a larval VG. A3) In the merged image, Ccap-gal4-driven mCD8GFP expression and TH immunoreactivity localized to different neuron groups. A4) Idealized schematic representation of the respective neuron groups in the Fas2 landmark system. B1-3) Corresponding transversal views of the neuromere a1 (* in A3), and B4) the deduced idealized scheme. In the schemes, all Ccap-gal4 x mCD8GFP expressing neurons lacked TH immunoreactivity and are shown in gray. Neurons which only showed TH immunoreactivity are blue-colored. Original images are maximum pixel intensity projections of volume-rendered 3D image stacks. Scale bars: 50 µm in A), 25 µm in B). (1.04 MB TIF) [file pone.0001848.s006.tif]
